# Characterization of the prognostic values and response to immunotherapy/chemotherapy of Krüppel‐like factors in prostate cancer

**DOI:** 10.1111/jcmm.15242

**Published:** 2020-04-13

**Authors:** Jialin Meng, Xiaofan Lu, Yujie Zhou, Meng Zhang, Lei Gao, Shenglin Gao, Fangrong Yan, Chaozhao Liang

**Affiliations:** ^1^ Department of Urology, The First Affiliated Hospital of Anhui Medical University Institute of Urology, Anhui Medical University Anhui Province Key Laboratory of Genitourinary Diseases Anhui Medical University Hefei China; ^2^ State Key Laboratory of Natural Medicines, Research Center of Biostatistics and Computational Pharmacy China Pharmaceutical University Nanjing China; ^3^ Division of Gastroenterology and Hepatology Key Laboratory of Gastroenterology and Hepatology Ministry of Health Renji Hospital School of Medicine Shanghai Institute of Digestive Disease Shanghai Jiao Tong University Shanghai China; ^4^ Department of Urology The Second Hospital of Hebei Medical University Shijiazhuang China; ^5^ Department of Urology The Affiliated Changzhou No. 2 People's Hospital of Nanjing Medical University Changzhou China

**Keywords:** chemotherapy, genetic alteration, immunotherapy, Krüppel‐like factor, promoter methylation, prostate cancer, recurrent‐free survival, transcription expression

## Abstract

At present, the overall genetic and epigenetic effects of Krüppel‐like factors (KLFs) on prostate cancer (PCa) remain unclear. Therefore, we systematically investigated the molecular differences in KLFs of transcription expression, promoter methylation and genetic alteration. Univariate and multivariate Cox proportional hazard regression was used to analyse the effect on RFS and establish the prognostic signature in the TCGA cohort, MSKCC and GSE116918 cohorts employed to validate the signature. Biological pathway enrichment and the potential response to immunotherapy and chemotherapy were inferred. The transcription levels of most KLFs are associated with the clinical outcome of PCa. Gleason score (*P* = .009), pathology T stage (*P* = .006), KLF3 (*P* = .034), KLF5 (*P* = .002) and KLF7 (*P* = .035) were independent prognostic factors. A prognostic signature was established in the TCGA cohort (*P* < .001) and validated in the MSKCC (*P* < .001) and GSE116918 cohorts (*P* = .006). Demethylation of KLF5 by 5‐azacytidine led to increased protein levels, whereas knockdown of KLF5 promoted cell proliferation. Patients in KLF‐F were more likely to respond to immunotherapy (*P* < .001) and bicalutamide (*P* < .001). In summary, we found that the KLFs and clinical feature‐based signatures may improve prognosis prediction in PCa and further promote patient stratification and disease management.

## 1 INTRODUCTION

Prostate cancer (PCa) is a common malignant carcinoma among males worldwide, and accounts for the second greatest prevalence and a fifth of cancer‐specific deaths. More than 300 000 deaths are caused by PCa annually, accounting for approximately 6.6% of cancer‐specific mortality in males.^1^, ^2^, ^3^ Additionally, PCa is the second most frequent cancer (13%) in the oldest‐old males, of whom older than 85 years old, and is the primary cause of mortality in the United States (20%).^4^ In China, the incidence of PCa has risen sharply from 10% to 20% over the last two decades due to the widespread use of prostate biopsies for diagnosis.^5^, ^6^, ^7^ The overall survival rate for patients with PCa is not poor compared with that of other malignancies, with more than 80% survival during the first five years after diagnosis.^8^, ^9^, ^10^ However, the recurrence rate of PCa is high, and most patients will enter the advanced castration‐resistant PCa (CRPC) stage, which increases the risk of PCa‐specific death.^11^, ^12^, ^13^, ^14^

Krüppel‐like factors (KLFs) are zinc finger proteins that bind to the DNA transcriptional region and act as transcriptional activators or repressors. Numerous biological processes are affected by KLFs, such as cell proliferation and differentiation as well as the development of mammalian tissues and organs by maintaining the homeostasis of both tissues and systemics.^15^, ^16^ There are 18 KLF family members, and each consists of three common conserved Cys2/His2 zinc fingers. KLF family members bind to similar promoter regions in the C‐terminal domains of genes, such as CACCC‐, GC or GT‐box.^17^ The features of KLFs are both exclusive and overlapping. For example, KLF2, KLF4 and KLF6 are involved in the activation of macrophages, and KLF4 induces the M2 phenotype macrophage through IL‐4, while KLF2 can inhibit NF‐κB‐dependent proinflammatory activation and promote M2 polarization.^18^, ^19^ In contrast, KLF6 promotes the polarization of M1‐type macrophages through the inhibition of the NF‐κB pathway.^20^, ^21^ Moreover, KLF2, KLF4 and KLF5 have been linked to the pluripotency of stem cells.^22^

**Figure 1:**
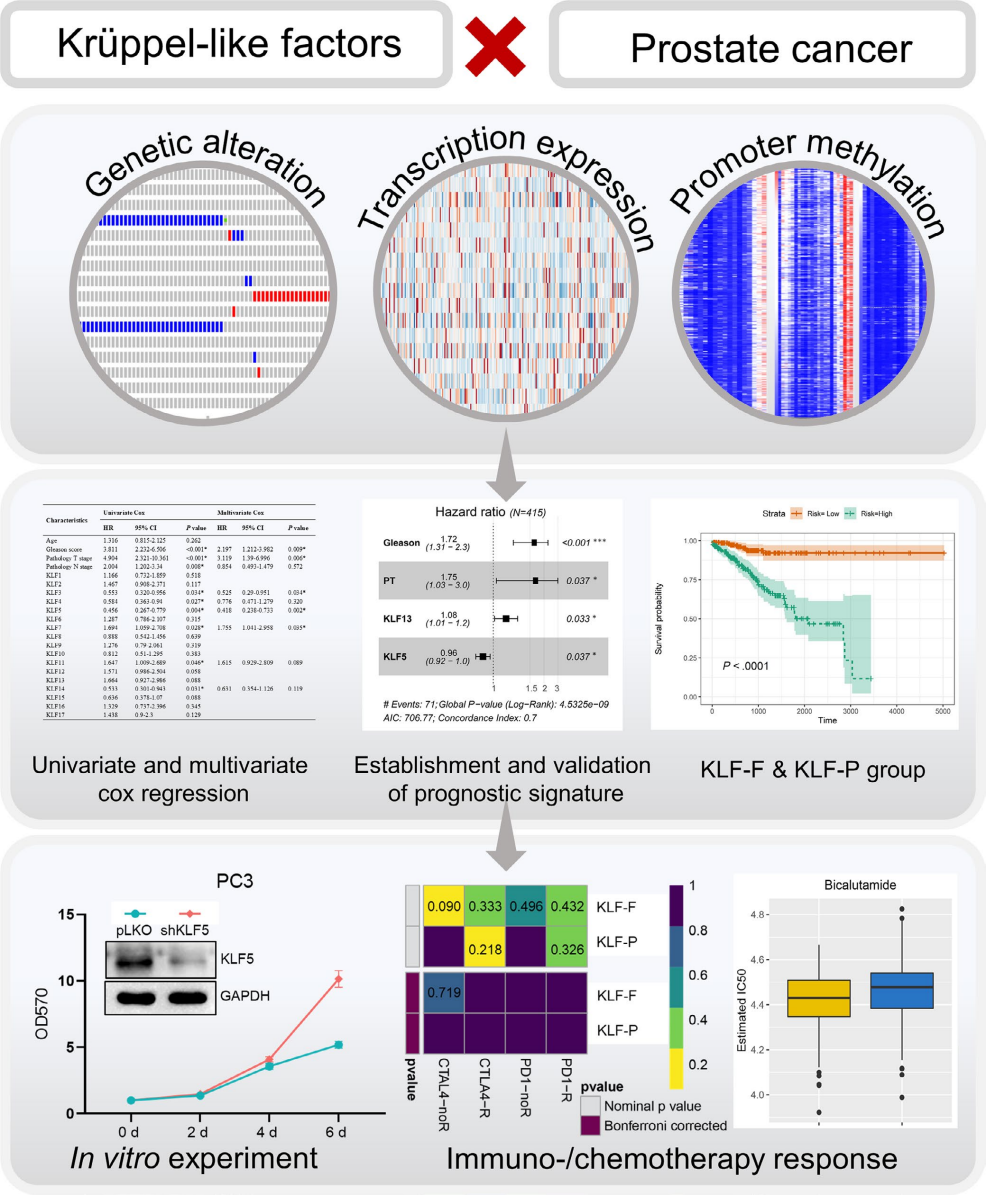
Flow chart illustrating the current study

In past decades, KLFs have been found to play pivotal roles in tumorigenesis. Kim et al found that KLF12 promotes tumour growth by directly activating EGFR and serves as a prognostic marker in colorectal cancer.^23^ Tsompana et al^24^ found that KLF4 targeted super‐enhancers and sustained the oncogenic state in head and neck squamous cell carcinoma. Jia et al found that the oncogenic role of KLF5‐regulated RP1 was accomplished through the suppression of p27kip1. However, the overall genetic and epigenetic effects of KLFs on PCa have not been investigated, until now. Therefore, in this study, we evaluated the prognostic values of KLF family members from different levels, including transcriptional expression, genetic alteration, DNA methylation and the likelihood of responding to immunotherapy or chemotherapy. The flow chart for the current study is depicted in Figure 1.

## 2 MATERIALS AND METHODS

### 2.1 Study population

We obtained the molecular data of PCa patients from The Cancer Genome Atlas Project (TCGA). The transcriptional expression data were acquired from the public data hub UCSC Xena (https://xenabrowser.net/), and consisted of 499 PCa samples and 52 normal samples. The transcriptomic profiles of the KLFs were extracted from the whole gene transcription data under the archive of the PRAD project. The MSKCC and GSE116918 cohorts were also enrolled as validation cohorts to evaluate the prognostic value of KLFs plus clinical feature signatures. The clinicopathological information for the enrolled cohorts is summarized in Table S1.

### 2.2 DNA methylation of KLFs

The β value ranges from 0 (unmethylated) to 1 (fully methylated), which indicates that the overall methylation level of the promoter region of KLFs was retrieved from MethHC (http://methhc.mbc.nctu.edu.tw/php/index.php).^25^ The correlation between the promoter methylation β value and KLFs expression level was evaluated via linear regression using GraphPad Prism 8.

### 2.3 Genetic alterations in KLFs

The genetic alterations of KLFs in patients with PCa were illustrated via the cBioPortal platform (http://www.cbioportal.org/),^26^, ^27^ which recorded the missense and truncating mutations as well as amplification and deep deletion. The RFS between patients with or without alterations was also conducted to evaluate the prognostic value of genetic alterations in KLFs. The expression of KLFs with gene amplification was depicted by GraphPad Prism 8.

### 2.4 Cell culture and reagents

The PC3, 22RV1, and HEK293T cell lines were purchased from the American Type Culture Collection (ATCC, Manassas, VA). HEK293T cells were cultured in DMEM medium, while PC3 and 22RV1 cells were cultured in RPMI 1640 medium. To prepare the media, 10% foetal bovine serum and 1% penicillin and streptomycin solution were added before use. The cell incubators were maintained at 37°C and 5% CO_2_. Bicalutamide (Sigma, #90357‐06‐5, ≥98%) was used at 10 μM for the treatment of 22RV1 cells.

### 2.5 Knockdown KLF5 plasmid design, lentivirus packaging and cell transfection

The primers used to generate the knockdown sequence of KLF5 were as follows: shKLF5‐forward: CCGGCCTATAATTCCAGAGCATAAAGGATCCTTTATGCTCTGGAATTATAGGTTTTTG, shKLF5‐reverse: AATTCAAAAACCTATAATTCCAGAGCATAAAGGATCCTTTATGCTCTGGAATTATAGG. After annealing and amplification, the newly generated ~60 bp sequence was inserted into the pLKP.1 plasmid to obtain the pLKO.1‐shKLF5 plasmid.

To obtain the knockdown KLF5 and control lentivirus, the psPAX2 packaging plasmid and pMD2.G envelope plasmid were cotransfected with the pLKO.1‐shKLF5 or plKO.1 plasmid into HEK293T cells. After 48 hours of incubation, the medium with excreted lentivirus was collected and stored at −80°C for infecting PCa cells.

### 2.6 MTT assay for cell growth and bicalutamide sensitivity

MTT assays were employed to evaluate cell proliferation. Pre‐prepared pLKO or shKLF5 PCa cells were digested and resuspended in medium, then seeded in 24‐well plates with 5000 cells in each well. The results were collected after 0, 2, 4 and 6 days. To evaluate bicalutamide sensitivity, 10 000 cells were seeded in several 24‐well plates and treated with or without 10 μM bicalutamide in pLKO or shKLF5 groups for four days. After collecting the results, 50 μL of MTT reagent (Amresco Inc, Solon) was added to the medium and incubated at 37°C for 2 hours. Afterwards, the medium was removed and 200 μL of dissolving reagent DMSO (Amresco Inc) was added to dissolve the formazan crystals. The optical density value was determined at a wavelength of 570 nm on a microplate reader.

### 2.7 Colony formation assay

pLKO and shKLF5 PCa cells (PC3 and 22RV1) were seeded in six‐well plates containing 800 cells per well and allowed to grow for an additional 12 days. Then, the culture solution was discarded, and the cells were rinsed twice with cold PBS. They were then fixed using 4% paraformaldehyde for 20 min and subsequently stained with 0.5% crystal violet staining solution for 20 min. The colonies were photographed and counted under a microscope.

### 2.8 Western blot

Cells were washed twice with cold PBS and lysed in RIPA lysis buffer, and proteins (40‐50 μg) were separated on 6%‐10% SDS/PAGE gels then transferred onto PVDF membranes (Millipore). After PVDF membranes were blocked, they were sequentially incubated with primary antibodies, HRP‐conjugated secondary antibodies, and visualized using an ECL system (Thermo Fisher Scientific). The primary antibodies used in the Western blot study included KLF5 (ABclonal, #A12403), E‐cadherin (ABclonal, #A11492), vimentin (ABclonal, #A2666) and GAPDH (Santa Cruz, #sc‐166574). The following day, anti‐rabbit, antimouse or anti‐goat IgG secondary antibody was used for 1 hour at a concentration of 1:5000 at 16°C and rinsed for 5 minutes with TBST three times.

### 2.9 Identification of risk‐associated differentially expressed genes (DEGs) and genome enrichment

The R package ‘edgeR’ was utilized to perform differential expression analysis with the standard comparison mode. DEGs were identified as genes that passed the threshold of *P* < .05 and absolute log2 fold‐change >0.5. The expression levels of the top DEGs for each patient were displayed with a heatmap, and GEPIA (http://gepia.cancer-pku.cn) was used to investigate their association with patient RFS.^28^ The DEGs were enrolled to generate the gene ontology (GO) and Kyoto Encyclopedia of Genes and Genomes (KEGGs) enrichment analysis using Metascape (http://metascape.org)^29^ with a threshold of false discovery rate <0.05. GSEA was used to identify pathways enriched in high‐ and low‐risk patient groups.

### 2.10 Immune infiltration, immunotherapy and chemotherapy response prediction

The infiltration of 22 subtypes of tumour‐infiltrating immune cells (TIICs) was retrieved from our previous study, which was calculated by CIBERSORT, an algorithm that quantifies the proportion of TIICs with 547 signature genes.^30^, ^31^ To evaluate the individual likelihood of responding to immunotherapy (eg immune checkpoint blockade), the Tumour Immune Dysfunction and Exclusion (TIDE) algorithm was employed.^32^, ^33^, ^34^ As immune checkpoint inhibitors have not yet been approved as routine drugs for PCa, subclass analysis was performed in response to anti‐CTAL‐4 or anti‐PD‐1 therapy based on the treatment results of 47 patients with melanomas who underwent immunotherapy.^35^

We also evaluated the chemotherapy response of each patient using the public pharmacogenomics database Genomics of Drug Sensitivity in Cancer (GDSC; https://www.cancerrxgene.org). Chemotherapy drugs cisplatin, docetaxel and bicalutamide that are normally used to treat patients with PCa were selected for evaluation. Based on the GDSC data, the half‐maximal inhibitory concentration (IC_50_) was estimated and represented the response of the drug. Therefore, the R package ‘pRRophetic’ was used with 10‐fold cross‐validation and other parameters by default.^36^

### 2.11 Statistics

To obtain the normalized expression data of KLFs, we converted the raw count data to the number of fragments per kilobase of non‐overlapped exon per million fragments mapped (FPKM). Survival analysis was performed using the R package ‘survival’. A Kaplan‐Meier (K‐M) curve was generated for survival rates of patients with different detections of the log‐rank test. The best cut‐off values were selected to divide the different survival groups, which were determined by algorithms embedded in the R package. The final prognostic K‐M plots are presented with a hazard ratio (HR), 95% confidence interval (CI) and log‐rank *P* value.

Univariate and multivariate Cox hazard proportional regression analyses of KLF members and clinical features were performed in RFS with a HR and 95% CI. The KLFs and clinical features based on the RFS predicting signature were constructed using the co‐efficient value derived from the Cox hazard proportional regression model. The risk score (ie signature) of each patient with PCa was calculated using a linear combination of mRNA expression of KLFs and clinical features, weighted by the corresponding coefficients and divided into favourable (KLF‐F) and poor (KFL‐P) RFS groups by the mean value of the signature. K‐M plots present the different RFS in the two groups, and the receiver operating characteristic (ROC) curve was plotted to illustrate the predictive performance of the signature. For all statistical analyses, *P* < .05 was considered statistically significant.

## 3 RESULTS

### 3.1 KLF expression and related RFS in patients with PCa

**Figure 2:**
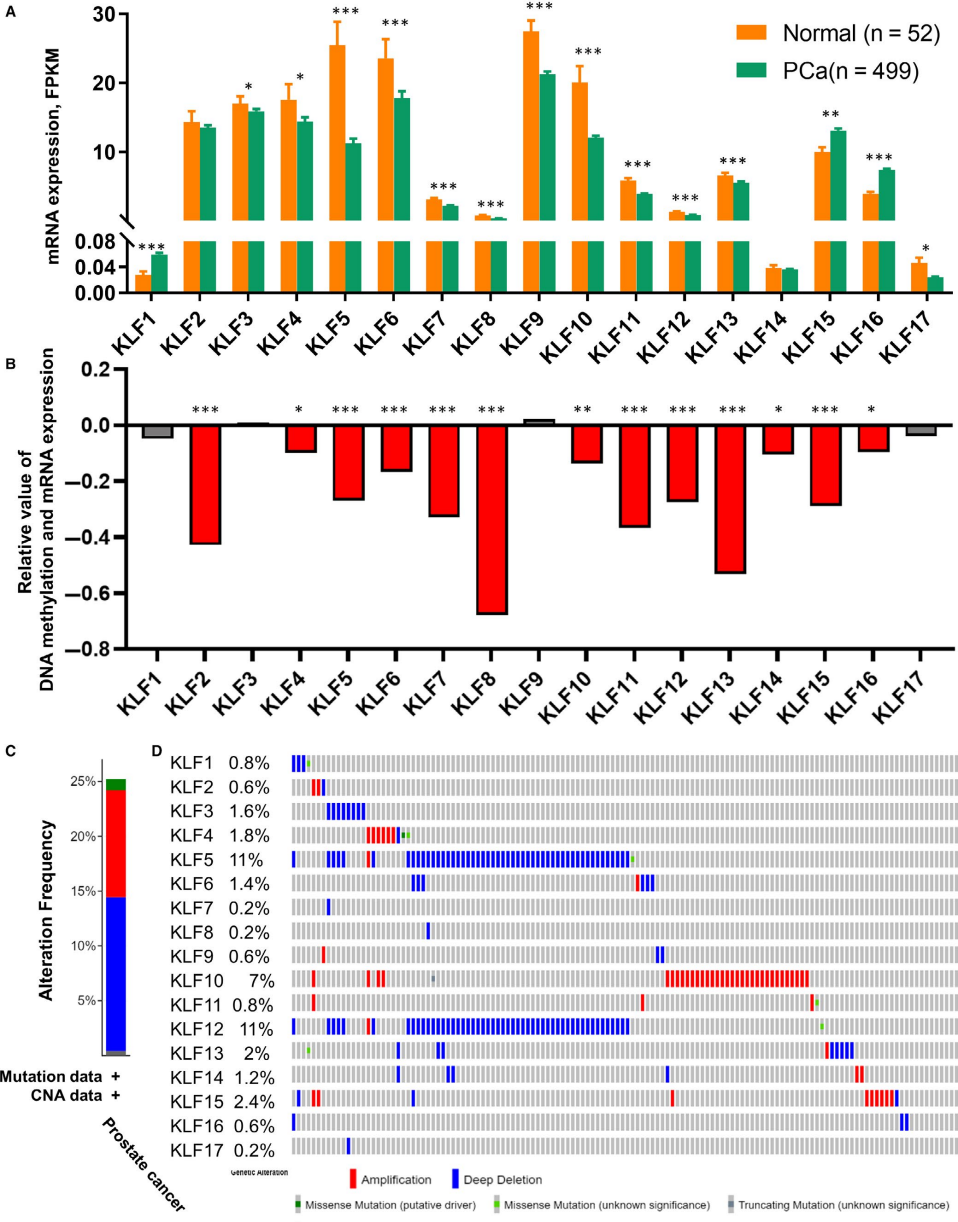
Transcriptional expression, DNA promoter methylation and genetic alteration of KLFs. A, The mRNA expression (FPKM) of KLFs between normal and PCa groups; B, the correlation between promoter methylation and mRNA expression of KLFs, *R* value indicates the linear relationship between DNA methylation and mRNA expression; C, the proportion of different types of genetic alteration to overall KLFs; D, the frequency and distribution of genetic alteration to each KLF; *P < .05, **P < .01, ***P < .001

We obtained and calculated the mRNA expression of 17 KLFs from the TCGA‐PRAD database, which contains 52 normal prostate tissues and 499 PCa tissues, while the mRNA level of KLF18 was not detectable and, thus, excluded from this study. As shown in Figure 2A, the mRNA expressions of the KLFs were polarized; most KLF levels decreased in PCa tissue compared with in normal tissue, including KLF3, KLF4, KLF5, KLF6, KLF7, KLF8, KLF9, KLF10, KLF11, KLF12, KLF13 and KLF17 (*P* < .05). In contrast, KLF1, KLF15 and KLF16 levels increased in tumour tissue (*P* < .05) and no significant difference was observed between the normal and tumour tissues with either KLF2 or KLF14 (Table S2). Furthermore, we evaluated the prognostic value of each KLF and found that the high expression of KLF7 (*P* = .035, Figure S1D), KLF11 (*P* = .024, Figure S1E) and KLF17 (*P* = .020, Figure S1H) indicated a poor prognosis with RFS, while the lower expression of KLF3 (*P* = .015, Figure S1A), KLF4 (*P* = .007, Figure S1B), KLF5 (*P* = .002, Figure S1C), KLF14 (*P* = .023, Figure 2F) and KLF15 (*P* = .033, Figure S1G) was associated with a favourable RFS. Prognostic analyses of other KLFs were also conducted using the K‐M curve, as shown in Figure S2.

### 3.2 KLF promoter methylation and genetic alteration

mRNA expression is affected by promoter methylation in cells; thus, we evaluated the promoter methylation levels of KLFs. We found that increased promoter methylation in the tumour tissues of KLF3, KLF6, KLF7, KLF8, KLF10, KLF11, KLF12 and KLF13 resulted in decreased mRNA expression as compared with normal tissues (*P* < .05; Table S3). We also evaluated the association between DNA methylation and mRNA expression of KLFs in PCa tumour tissues and found that the mRNA expression of most KLFs was negatively associated with its DNA methylation at the promoter region (*P* < .05), except for KLF3, KLF9 and KLF17 (Figure 2B).

Genetic alteration is another critical factor that affects the mRNA expression of genomic genes. Appropriately, 15% deep deletion occurred among TCGA‐PRAD patients to all KLFs, while amplification accounted for approximately 10%, and the genetic mutation fraction was less than 5% (Figure 2C). What is more, we identified a profound deep deletion rate in KLF5 (11%) and KLF12 (11%), while KLF10 had the highest amplification rate among all KLFs (7%; Figure 2D). Furthermore, this study revealed that the deletion of KLF5 (heterozygous deletion vs. homozygous deletion: *P* = .0103; Diploid vs. homozygous deletion: *P* = .0085; Diploid vs. heterozygous deletion: *P* = .0001; Figure S3A) and KLF12 (heterozygous deletion vs. homozygous deletion: *P* = .0377; Figure S3B) affected their mRNA levels. As the mRNA levels of KLFs are associated with a poor prognosis, genetic alteration and DNA methylation might be involved.

### 3.3 Univariate and multivariate analyses of KLFs and prognostic value

To evaluate the effect of KLFs and clinical features on the prognosis of PCa and to identify the positively associated factors, we conducted univariate and multivariate Cox regression analyses; the results are summarized in Table 1. In the univariate cox regression, we found that the Gleason score (HR = 1.977, 95% CI: 1.537‐2.541, *P* < .001), pathology T stage (HR = 2.597, 95% CI: 1.641‐4.109, *P* < .001), N stage (HR = 2.005, 95% CI: 1.203‐3.341, *P* = .008), KLF5 (HR = 0.957, 95% CI: 0.921‐0.995, *P* = .027) and KLF13 (HR = 1.089, 95% CI: 1.016‐1.167, *P* = .016) are associated with the RFS of patients with PCa. Due to low expression levels, we excluded KLF1, KLF14 and KLF17 when conducting univariate Cox regression analysis. Subsequently, we adopted multivariate Cox proportional hazard regression analysis based on the nine positive factors, and demonstrated that the Gleason score (HR = 1.723, 95% CI: 1.301‐2.282, *P* < .001), pathological T stage (HR = 1.754, 95% CI: 1.03‐2.985, *P* = .038), KLF5 (HR = 0.96, 95% CI: 0.925‐0.998, *P* = .038) and KLF13 (HR = 1.08, 95% CI: 1.006‐1.159, *P* = .033) are independent prognostic factors of PCa (Table 1).

**Table 1:** The univariate and multivariate analysis of RFS to KLFs and clinical‐pathological data from TCGA‐PRAD cohort

Characteristics	Univariate Cox			Multivariate Cox		
	HR	95% CI	*P* value	HR	95% CI	*P* value
Age	1.316	0.815‐2.126	.261			
Gleason score	1.977	1.537‐2.541	<.001*	1.723	1.301‐2.282	<.001*
Pathological T stage	2.597	1.641‐4.109	<.001*	1.754	1.03‐2.985	.038*
Pathological N stage	2.005	1.203‐3.341	.008*	0.983	0.567‐1.707	.952
KLF2	1.013	0.992‐1.034	.226			
KLF3	0.975	0.937‐1.015	.217			
KLF4	0.973	0.944‐1.002	.065			
KLF5	0.957	0.921‐0.995	.027*	0.96	0.925‐0.998	.038*
KLF6	0.987	0.968‐1.007	.206			
KLF7	1.144	0.903‐1.45	.265			
KLF8	1.277	0.44‐3.707	.653			
KLF9	1.003	0.977‐1.029	.846			
KLF10	0.986	0.961‐1.012	.296			
KLF11	1.12	0.978‐1.282	.103			
KLF12	1.098	0.668‐1.806	.712			
KLF13	1.089	1.016‐1.167	.016*	1.08	1.006‐1.159	.033*
KLF15	0.988	0.951‐1.026	.522			
KLF16	1.012	0.942‐1.088	.737			

* *P* < .05.

### 3.4 Establishment and validation of KLF‐related prognostic signature

**Figure 3:**
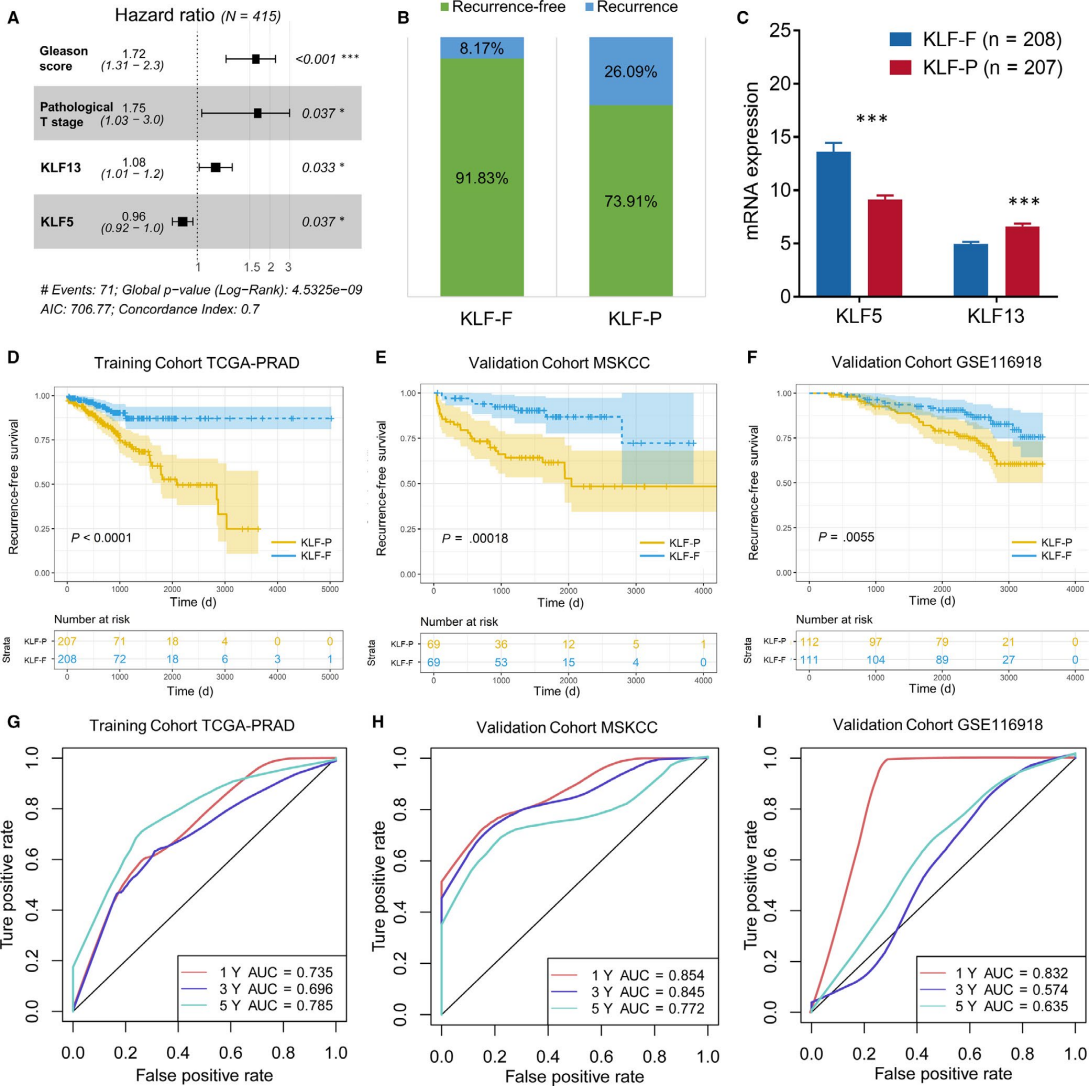
Establishment and validation of the prognostic signature of KLFs. (A) The hazard ratio of each independent risk factor to RFS; (B) the different distribution of biochemical recurrence in KLF‐F and KLF‐P groups; (C) mRNA expression of KLF5 and KLF13 between KLF‐F and KLF‐P groups, ***P < .001; K‐M plot to depict the different RFS in the TCGA‐PRAD training cohort (D), external validation MSKCC cohort (E) and GSE116918 cohort (F). The ROC curve and AUC value to assess the predictive value of the risk signature in the TCGA‐PRAD training cohort (G), external validation MSKCC cohort (H) and GSE116918 cohort (I)

To further explore whether and how KLFs impact the process and prognosis of PCa, we calculated the risk score for each patient based on the following formula: risk score = 0.542*Gleason score + 0.559*pathology T stage −0.040*KLF5 + 0.077*KLF13 (Figure 3A, Table S4; see Section 2). In this formula, the Gleason score was divided into five groups with different scores. The pathological T stage included four groups: T1, T2, T3 and T4. With the risk score, patients were divided into KLF‐F (low‐risk, n = 208) and KLF‐P (high‐risk, n = 207) groups. The recurrence rate was high at 26.09% in the KLF‐P group, while only 8.17% of patients met the recurrence rate in the KLF‐F group (Figure 3B). We also evaluated the expression of KLF5 and KLF13 and identified the decreased expression of KLF5 in the KLF‐P group, along with an increased level of KLF13 (Figure 3C). Subsequently, the K‐M plot was employed to test the discrimination value of the risk score, as shown in Figure 3D. RFS was significantly different between the KLF‐P and KLF‐F groups (*P* < .001), and the ROC curve also demonstrated a favourable value of the risk score for predicting recurrence in the TCGA‐PRAD cohort (1‐year AUC = 0.735; 3‐year AUC = 0.696; 5‐year AUC = 0.785, Figure 3G).

To appraise the availability and stability of the KLF‐related prognostic signature, we used the same formula mentioned above to generate the risk score of each patient in the MSKCC and GSE116918 cohorts. The K‐M plot based on the MSKCC cohort showed poor RFS in the KLF‐P group (*P* = .00018, Figure 3E), and the AUC value illustrated a good quality of distinguishability in 1 year (AUC = 0.854), 3 years (AUC = 0.845) and 5 years (AUC = 0.772, Figure 3H). Results from the GSE116918 cohort also indicated a good application of the prognostic signature with a dramatic RFS difference between KLF‐F and KLF‐P groups (*P* = .0055, Figure 3F) as well as a high AUC value (1‐year AUC = 0.832; 3‐year AUC = 0.574; 5‐year AUC = 0.635, Figure 3I).

### 3.5 KLF5 expression affected by 5‐azacytidine and knockdown of KLF5 promoted cell proliferation of PCa

**Figure 4:**
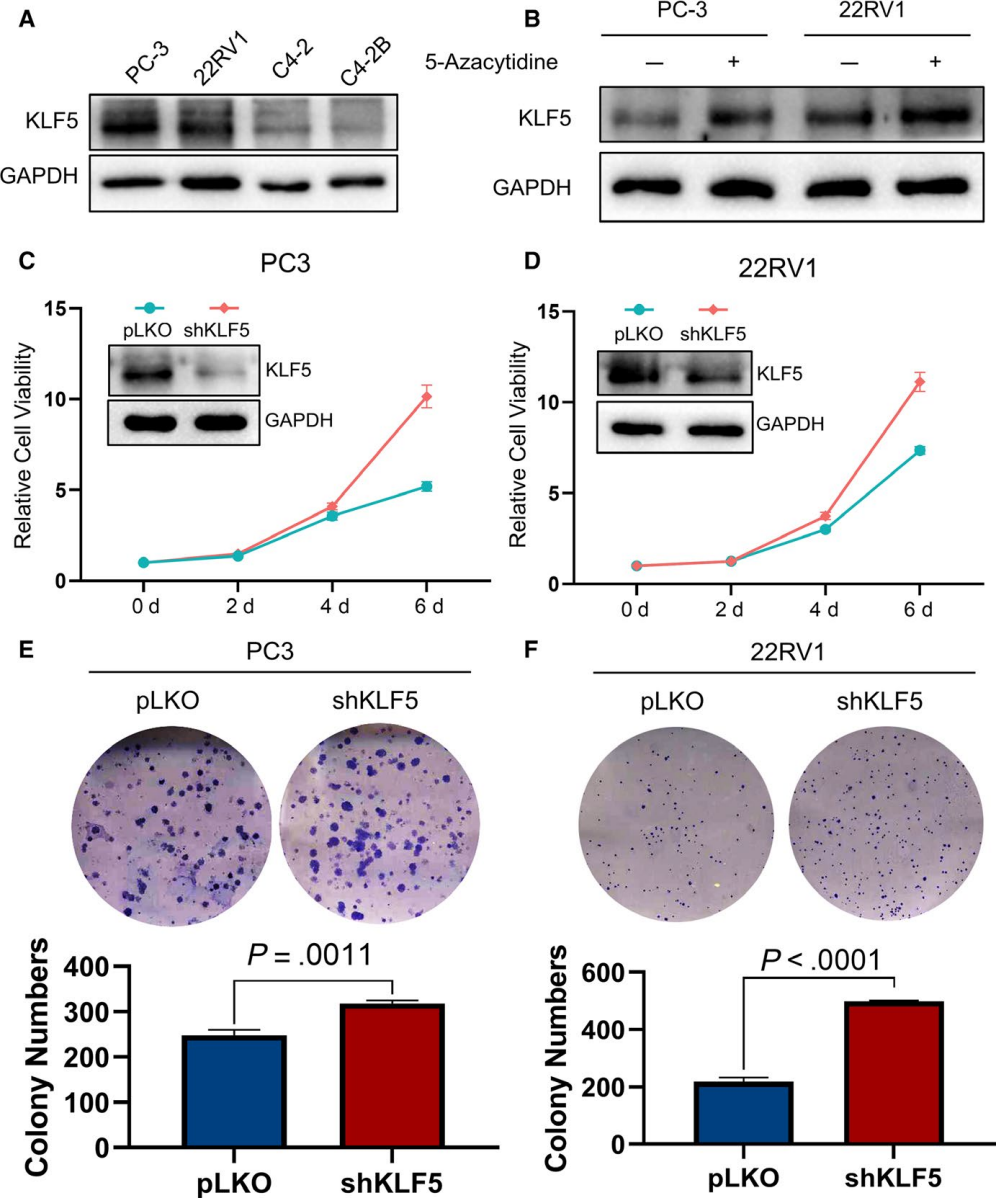
Knockdown of KLF5 promotes the cell proliferation of PCa. (A) Baseline protein levels of KLF5 in four PCa cell lines; (B) 5‐azacytidine inhibited DNA methylation and promoted the expression of KLF5 in PC‐3 and 22RV1 cells; KLF5 promotes cell proliferation in PC‐3 (C) and 22RV1 (D) cells, as assessed by MTT assay. Knockdown of KLF5 promotes cell proliferation in PC‐3 (E) and 22RV1 (F) cells, as assessed by the colony formation assay

We first detected the baseline KLF5 protein levels in several PCa cell lines and found that KLF5 is highly expressed in PC3 and 22RV1 cells and is lower in C4‐2 and C4‐2B cells (Figure 4A). Therefore, we selected PC3 and 22RV1 cell lines for the next validation. Based on the analysis between KLF expression and DNA methylation, we found that KLF5 is negatively associated with the methylation of its upstream promoter region (Figure 2B). Therefore, we detected the protein level of KLF5 after treatment with 5‐azacytidine, a commonly used inhibitor of DNA methylation.^37^ As shown in Figure 4B, after treatment with 5‐azacytidine, the protein levels of KLF5 increased in PC3 and 22RV1 cell lines. Subsequently, we used the shKLF5 lentivirus to knockdown KLF5 and found that cell proliferation significantly increased after knockdown of KLF5 in PC3 and 22RV1 cells (Figure 4C,D), and the same tendency was also observed in the colony formation assay (Figure 4E,F).

### 3.6 Identification of DEGs between KFL‐F and KLF‐P groups and pathway enrichment

**Figure 5:**
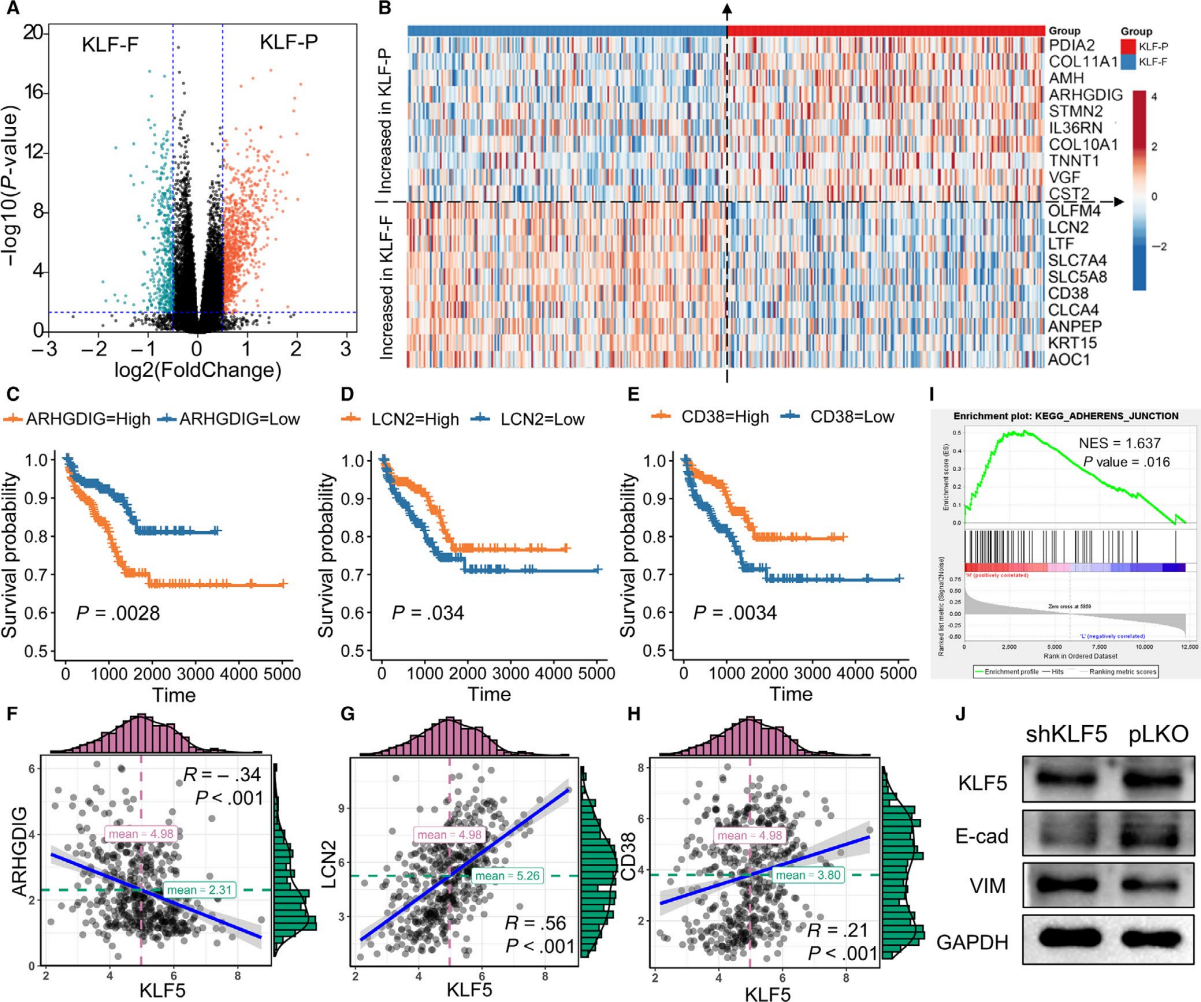
Potential biological pathways affected by KLFs. (A) The DEGs between the KLF‐F and KLF‐P groups; (B) top 10 increased and decreased genes among the KLF‐F and KLF‐P groups. Highly expressed ARHGDIG from the KLF‐P group predicted a poor prognosis (C) and its expression was negatively associated with KLF5 (F). Highly expressed LCN2 and CD38 from the KLF‐F group was linked with a favourable prognosis (D,E) and their expressions were positively associated with KLF5 (G,H). (I) The GSEA results showed that KEGG_ADHERENS_JUNCTION is activated in the KLF‐P group; (J) the EMT pathway is activated after knockdown of KLF5

The DEGs in the KFL‐F and KLF‐P groups were obtained using ‘edgeR’ R packages with log_2_ fold‐change >0.5 or <−0.5 and *P* value <0.05. The red dot represents the highly expressed gene in KLF‐P, while the blue dot represents the highly expressed gene in KLF‐F (Figure 5A). The top 10 highly expressed genes in the KFL‐F and KLF‐P groups are shown in Figure 5B. The highly expressed ARHGDIG in the KLF‐P group leads to a poor prognosis of PCa (*P* = .00028, Figure 5C), and the expression of ARHGDIG in tumours is negatively associated with the KLF5 levels (*R* = −0.34, *P* < .001, Figure 5F), confirming the prognostic value of KLF5 in PCa. Meanwhile, the high levels of the KLF‐F group highly expressed LCN2 and CD38 linked to a better RFS (*P* < .05, Figure 5D,E), and both were positively correlated with increased levels of KLF5 (LCN2: *R* = 0.56, *P* < .001; CD38: *R* = 0.21, *P* < .001, Figure 5G,H).

To obtain an in‐depth understanding of the association between and the prognosis of PCa, we performed functional enrichment analyses among the DEGs. We first used GSEA to analyse the different pathways enrolled in the KLF‐F and KLF‐P groups, and the results showed that the KEGG _ADEHERENS_JUNCTION pathway was highly activated in the KLF‐P group (NES = 1.637, *P* = .016, Figure 5I). The epithelial‐mesenchymal transition pathway is the pivotal pathway in the cell adherens junction; therefore, we evaluated the alteration of the EMT pathway and found that after the knockdown of KLF5 (simulating KLF‐P status), the protein levels of E‐cadherin decreased, while vimentin increased considerably. These WB results showed that the EMT pathway was activated after the knockdown of KLF5 (Figure 5J). We also used Metascape to generate the overall function of the different genes in the KLF‐F and KLF‐P groups in GO biological processes, reactome gene sets, KEGG pathways and canonical pathways. Figure S4A displays the pathway enrichment of highly expressed genes in KLF‐P. Enrichment is primarily related to nuclear division (red and green dots) and the cell cycle of mitotic cells (blue dot). For KLF‐F‐related enrichment, the NABA matrisome‐associated pathway (red dot) was mostly enriched, which affects the extracellular matrix. We found that the chemotaxis, tissue morphogenesis and second‐messenger‐mediated signalling pathways were also annotated in the KLF‐F‐associated gene group (Figure S4B).

### 3.7 Different immune infiltration between KFL‐F and KLF‐P groups

**Figure 6:**
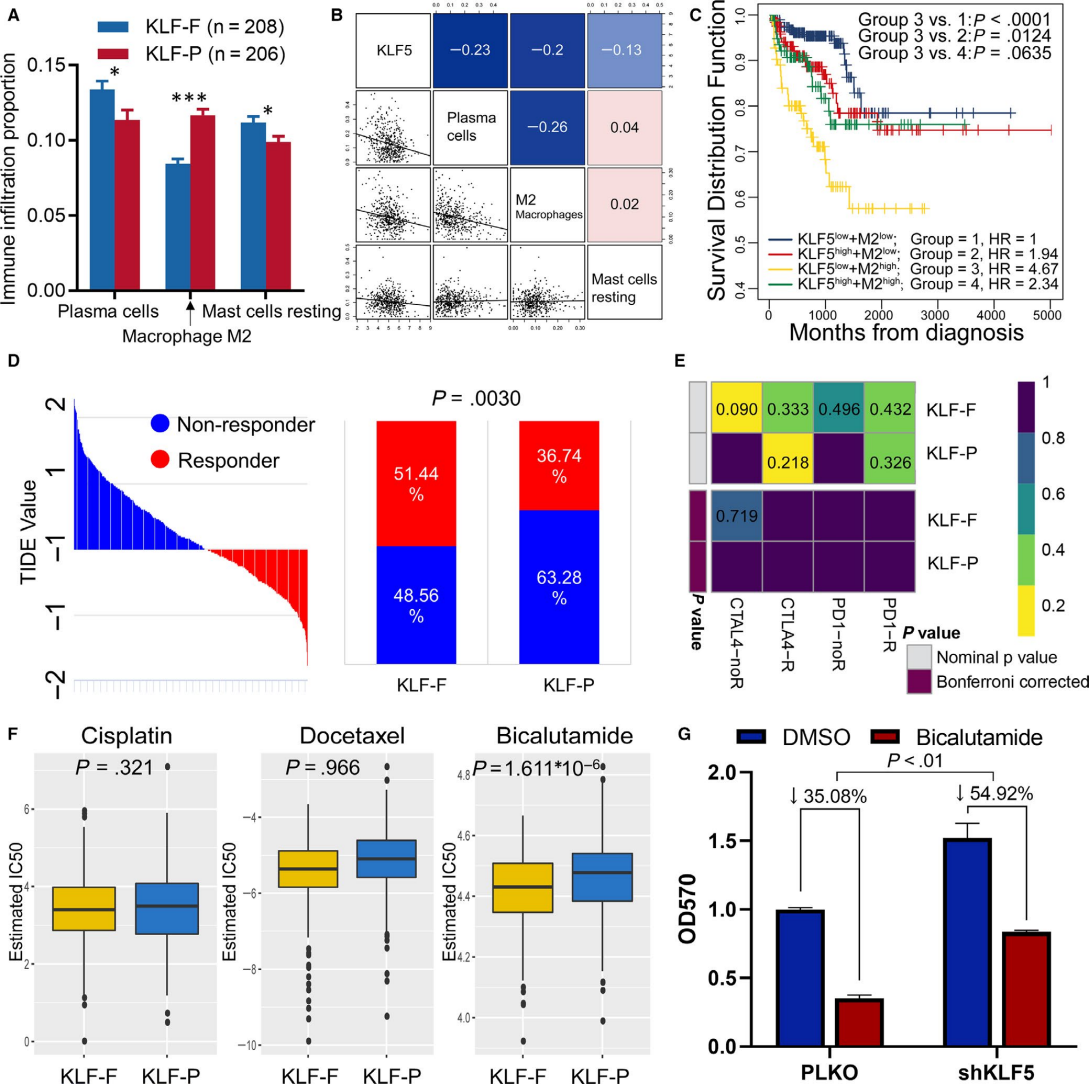
Immune infiltration and immunotherapy and chemotherapy among KLF‐F and KLF‐P subgroups. A, Immune infiltration proportion of TIICs between normal and PCa groups, *P < .05, ***P < .001; B, the correlation between KLF5 and the infiltration of plasma cells, M2 macrophages, and resting mast cells; C, K‐M plot showing the different RFS outcomes in KLF5 and M2 macrophage‐determined subgroups; D, the TIDE value and response results to immunotherapy of patients with PCa; E, Submap analysis showed no difference in response to CTAL‐4 and anti‐PD‐1 therapy; F, estimated IC_50_ indicates the efficiency of chemotherapy to KLF‐F and KLF‐P groups by cisplatin, docetaxel and bicalutamide; G, knockdown of KLF5 increased bicalutamide resistance to 22RV1 cells

The infiltration of TIICs in tumours plays a key role in the tumour environment and affects prognosis. In the present study, we found that KLKs distinguished patients with PCa with poor a prognosis (KLF‐P) or favourable prognosis (KLF‐F); thus, we further investigated the different TIIC infiltrations in tumours with different prognoses. We found that the distributions of plasma cells (*P* = .020) and resting mast cells (*P* = .024) were higher in KLF‐F, while M2 macrophage (*P* < .001) infiltration was higher in the KLF‐P than the KFL‐F group (Figure 6A, Table S5). Then, we evaluated the association between KLF5 expression and the above three immune cell infiltrations and found that plasma cells and M2 macrophages were negatively associated with KLF5 expression (Figure 6B). In our previous study, we revealed that the high infiltration of M2 macrophages is linked with the poor prognosis of patients with PCa, and this study revealed that KLF5 is the key gene against the progression of PCa in databases and in vitro. Therefore, we analysed the combined effect of KLF5 and M2 macrophages and found that patients with low KLF5 and high M2 macrophage infiltration had the worst prognosis (HR = 4.67, *P* < .001, Figure 6C).

### 3.8 Immunotherapy and chemotherapy are more practical for KLF‐F patients

We further assessed the potential response to immunotherapy in each patient using the TIDE algorithm (Table S6), and observed that patients in the KLF‐F group (51.92%, 108/208) were more likely to respond to immunotherapy than those in the KLF‐P group (36.23%, 75/207; *P* = .0015; Figure 6D). Subsequently, we analysed the response of anti‐CTLA‐4 and anti‐PD‐1 therapy; however, we could not conclude the different responses between the KLF‐F and KLF‐P groups regarding both immunotherapies (Figure 6E).

Chemotherapy is another method of treating advanced PCa in a clinical setting. Therefore, we focused on the different responses to chemotherapy of KLF‐associated KFL‐F and KLF‐P groups. The cell line data obtained from the GDSC database were employed to train the predictive model based on the satisfied predictive accuracy evaluated by 10‐fold cross‐validation. The IC_50_ values for cisplatin, docetaxel and bicalutamide of each patient with PCa were estimated by the predictive model. Finally, we recognized a dramatic difference in the estimated IC_50_ among KLF‐determined risk groups, of which the KLF‐F group showed a better response to bicalutamide (*P* < .001; Figure 6F) treatments.

## 4 DISCUSSION

PCa is a considerable worldwide burden on public health. The symptoms of urination discomfort, bone metastasis pain and castration treatment failure, and the high rates of recurrence make it an urgent public health event.^38^, ^39^ To identify the prognoses of patients with PCa, several studies have focused on establishing a prognostic signature. Zhao et al^40^ focused on the immune landscape and revealed that macrophages and T cells conferred the worse RFS, and suggested that PD‐L2 might be a potential therapeutic target for patients with PCa. Shao et al^41^ revealed a seven‐lncRNA signature to predict the RFS in PCa, which showed an AUC of 0.718 to three‐year RFS after adjustment for other major clinical features. Alshalalfa et al^42^ reported a 212 gene‐based SCGScore to identify the phenotype of neuroendocrine prostate carcinoma, which always produced an unfavourable prognosis.

KLF family members are a series of DNA transcriptional regions that bind zinc finger proteins and promote or suppress the transcriptional levels of downstream genes. KLFs are widely reported in the initial stages of tumorigenesis. Wang et al ^43^ reported that KLF2 directly regulated the expression of PTEN in gastric cancer and served as a downstream target of miR‐32‐5p, and that the decrease in KLF2 could promote gastric cancer development. Mao et al^44^ also illustrated the poor survival impact of KLF8 in gastric cancer, which regulated glycolysis by affecting GLUT4. Sun et al^45^ found that a low level of KLF3 is associated with the poor prognosis of lung cancer, and KLF3 could alter the epithelial‐mesenchymal transition by controlling STAT3, ultimately affecting metastasis. Hu et al^46^ investigated the effect of KLF13 in glioma, and found that the antibiotic clofoctol could suppress the proliferation of glioma stem cells by activating the expression of KLF13. Regarding PCa, Luo et al^47^ reported that KLF14 could regulate the antioxidant response and subsequent pathogenesis of CRPC through an HO‐1 adaptive mechanism. Shen et al^48^ and Wang et al^49^ reported that KLF9 and KLF13 could both suppress the growth of PCa by inhibiting the activation of AKT signalling. He et al^50^ obtained an optative view that KLF8 was overexpressed in PCa and promoted the proliferation of PCa cells by co‐activating the androgen receptor.

In the present study, we depicted a landscape of the prognostic value of KLFs from multiple aspects, including transcription, methylation, genetic alteration, potential signalling pathways and specific therapeutic methods. To leverage the complementary molecular and clinical characteristics, we integrated the molecular characteristics and clinical factors to build a composite prognostic signature consisting of KLF5, KLF13, Gleason score and pathology T stage from the TCGA cohort, and validated it in the MSKCC and GSE116918 cohorts. DNA methylation plays an important role in tumorigenesis. Yang et al^51^ reported that complex SUV39H1/CRL4B/HP1/DNMT3A promoted DNA methylation‐based epigenetic silencing, while Spyropoulou et al^52^ also showed that histone lysine N‐methyltransferases, especially SUV39H1, led to malignancy in gliomas and is a potential biomarker. In this study, the in vitro experiment confirmed the tumour suppression function of KLF5 in PC‐3 and 22RV1 cell lines, and the DNA methylation inhibitor demethylated and caused re‐expression of KLF5. Furthermore, we evaluated the different signalling pathways in KLF‐F and KLF‐P patients. GSEV analysis was employed and revealed that the KEGG_ADHERENS_JUNCTION pathway was activated in the KLF‐P group, and we validated the new funding in 22RV1 cells and the EMT pathway was significantly activated after the knockdown of KLF5.

Immunotherapy and chemotherapy are now widely used to treat different tumours. Recently, personalized treatment of cancer has been a focus of clinicians and scientists. For example, in a phase III trial of nivolumab for advanced non‐small cell lung cancer, patients with a high tumour mutation burden (TMB) had significantly improved PFS in the nivolumab group compared with the chemotherapy group, which means that patients with high TMB may benefit more from the treatment of nivolumab.^53^ Kijima et al^54^ also reported that patients with colorectal cancer who have a low expression of miR‐6826 may have a better response after vaccination, as well as miR‐6875. In the current study, we found that the critical KLFs along with clinical features divided patients into KLF‐F and KLF‐P, thus, we also appraised the distinctive response to immunotherapy and chemotherapy. Finally, we determined that KLF‐F patients had a better response to bicalutamide based on the results of database analysis and in vitro experiments, while immunotherapy showed no difference in KLF‐F and KLF‐P subgroups.

There are some advantages that should not be neglect in the current study. We established and validated a novel KLF‐associated prognostic signature to help predict outcomes among almost 776 patients with PCa. Therefore, clinicians could use the signature to predict underlying recurrence and carry out effective treatment. In addition, the molecular function of KLF5 was confirmed in PCa cell lines, which could affect the proliferation and treatment sensitivity of bicalutamide through the EMT pathway. Meanwhile, some limitations should also be addressed and modified in future studies. First, more clinical samples should be used to confirm the effectiveness of the KLF‐associated prognostic signature. Second, although we found that the KLF‐F patients could benefit more from immunotherapy, there is no difference between KLF‐F and KLF‐P patients in CAR‐T and PD‐1/PD‐L1 therapy; thus, the potential novel immunotherapy should be investigated in future. Third, the potential mechanism by which KLF5 promotes cell proliferation and affects bicalutamide sensitivity should be studied.

In summary, KLF family members are essential prognostic factors for PCa. The KLFs and clinical feature‐based signatures identified the unfavourable prognosis precisely, while Bicalutamide is an effective medicine to treat KLF‐F patients.

## CONFLICT OF INTEREST

The authors declare that they have no conflict of interest.

## AUTHORS' CONTRIBUTIONS

Jialin Meng, Xiaofan Lu and Shenglin Gao conceived and designed the research; Yujie Zhou and Meng Zhang performed the date record and collection; Jialin Meng, Lei Gao and Yujie Zhou conducted the data analysis and visualization; Fangrong Yan, Chaozhao Liang and Shenglin Gao wrote and review the manuscript. All authors read and approved the final manuscript.

## Supporting information

Supplementary materials

## Data Availability

The data sets used and/or analysed during the current study are available from the corresponding author on reasonable request.
