# Supplementary material for: Characterization of the prognostic values and response to immunotherapy/chemotherapy of Krüppel‐like factors in prostate cancer
Source: J Cell Mol Med. 2020 Apr 13;24(10):5797–810. doi: 10.1111/jcmm.15242 (PMC7214179; doi:10.1111/jcmm.15242)
Supplement: Supplementary file 1 — Supplementary materials [file JCMM-24-5797-s001.pdf]

Supplementary figures and tables

Figure S1. K-M plots to shown the positive RFS difference in high and low level of KLFs.

Figure S2. K-M plots to shown the negative RFS difference in high and low level of KLFs.

Figure S3. The genetic amplification effect on mRNA expression of KLF5 and KLF12.

Figure S4. The pathway enrichment of increased genes in KLF-P and KLF-F groups.

Table S1. The clinicopathological information of training and validation cohorts.

Table S2. The mRNA expression of KLF family members among normal and tumor tissues.

Table S3. The promoter methylation of KLF family members among normal and tumor tissues.

Table S4. The coefficient of KLFs and clinical features conducted by the Cox proportional hazard regression analyses.

Table S5. The distribution of TIICs among KLF-F and KLF-P group.

Table S6. The result of response to immunotherapy based on TIDE algorithm method.

Figure S1. K-M plots to shown the positive RFS difference in high and low level of KLFs

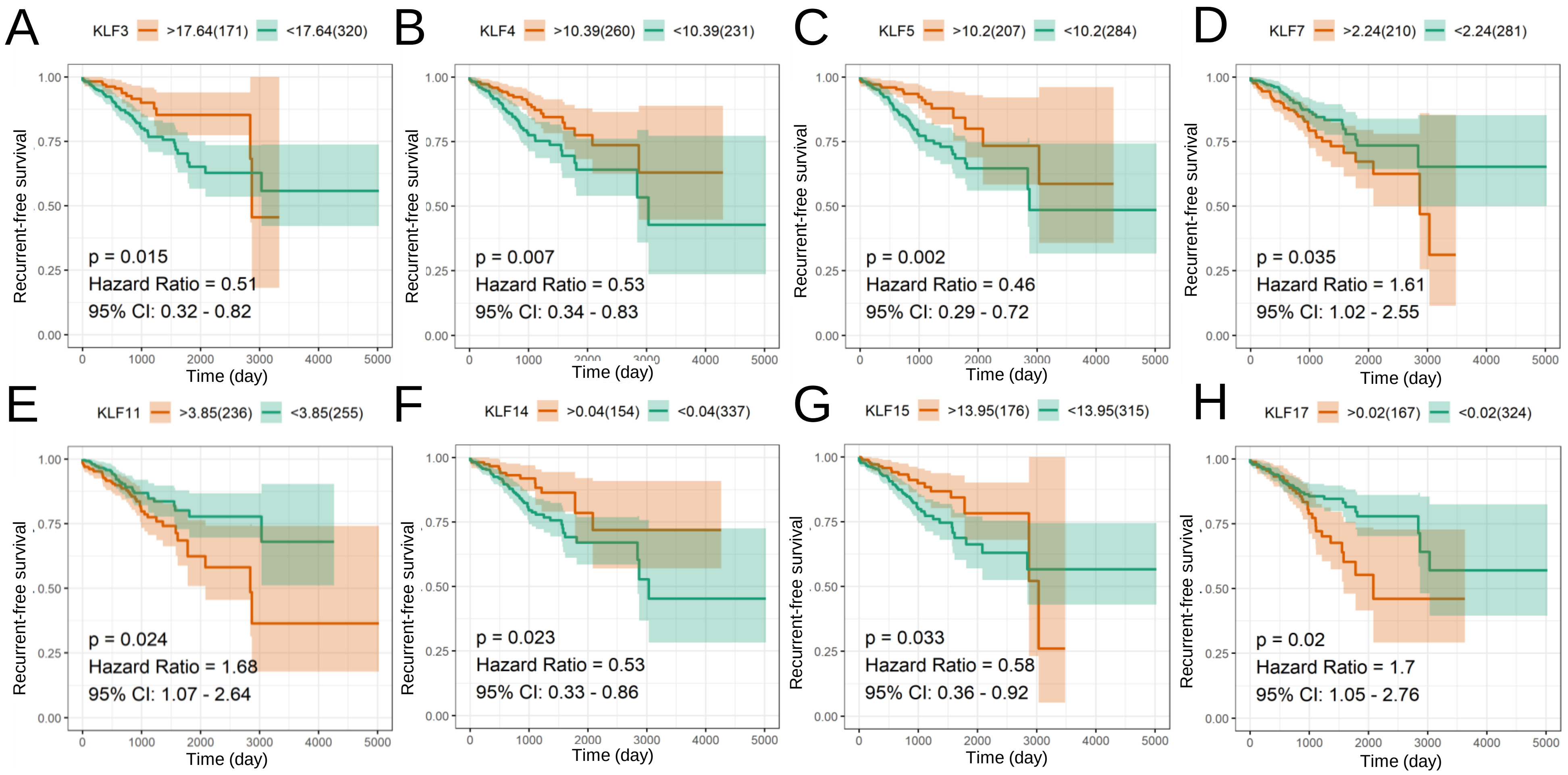

Figure S2. K-M plots to shown the negative RFS difference in high and low level of KLFs

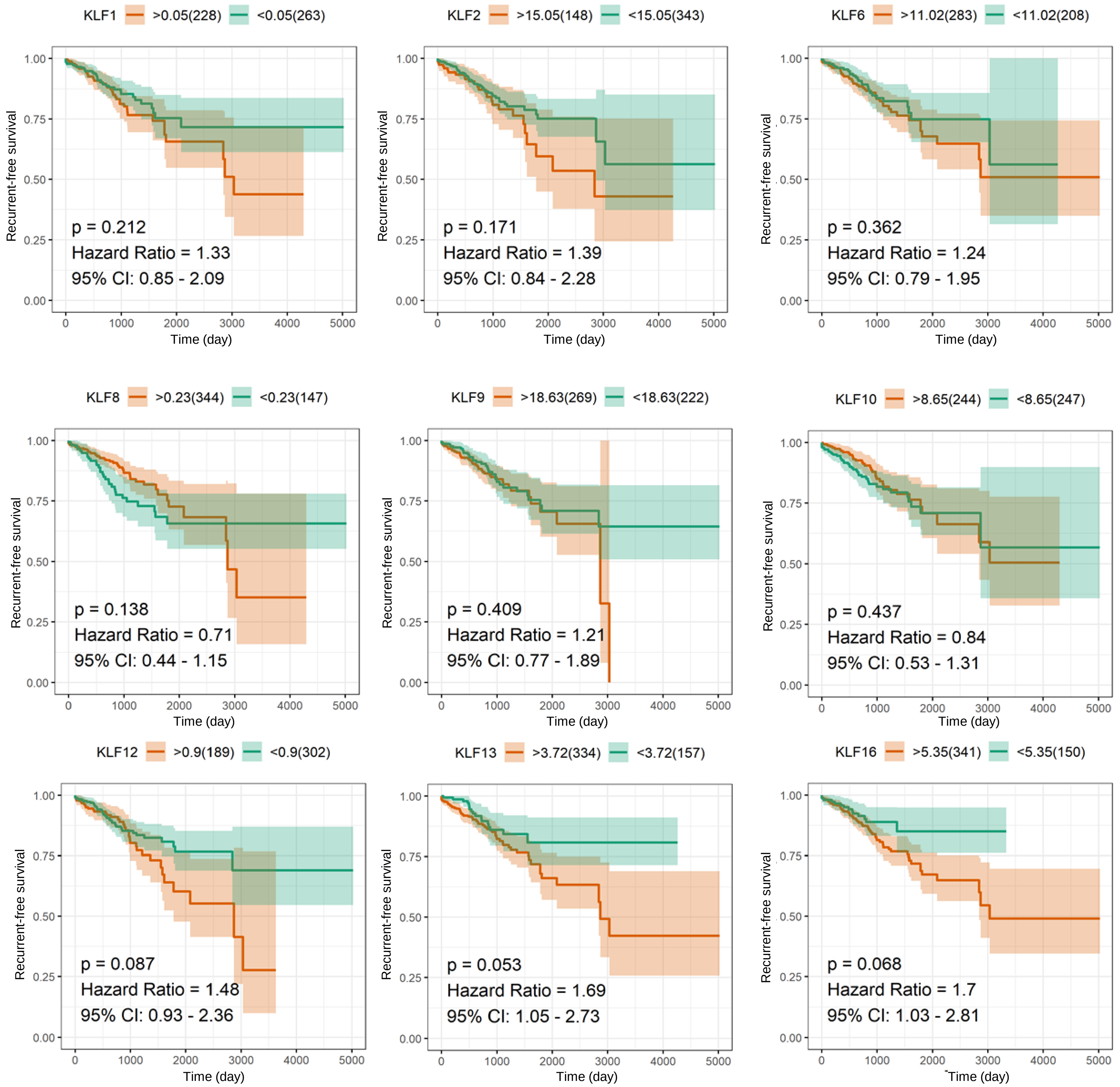

Figure S3. The genetic amplification effect on mRNA expression of KLF5 and KLF12

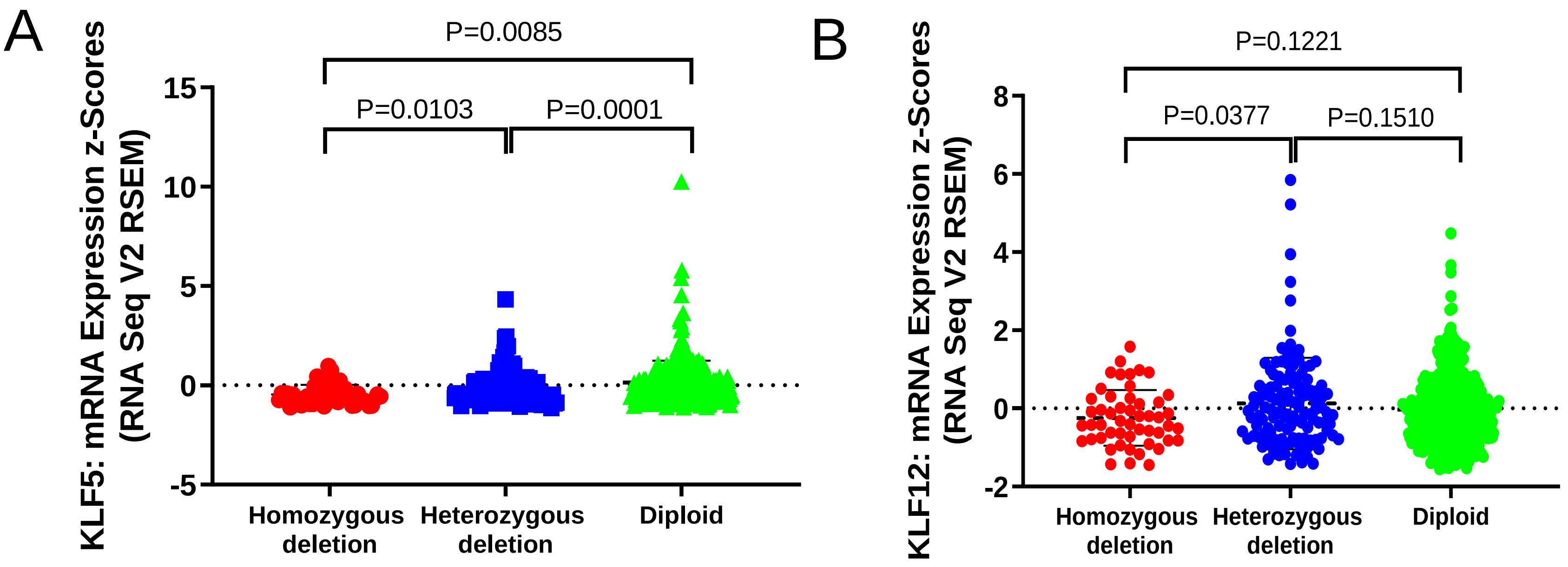

Figure S4. The pathway enrichment of increased genes in KLF-P and KLF-F groups.

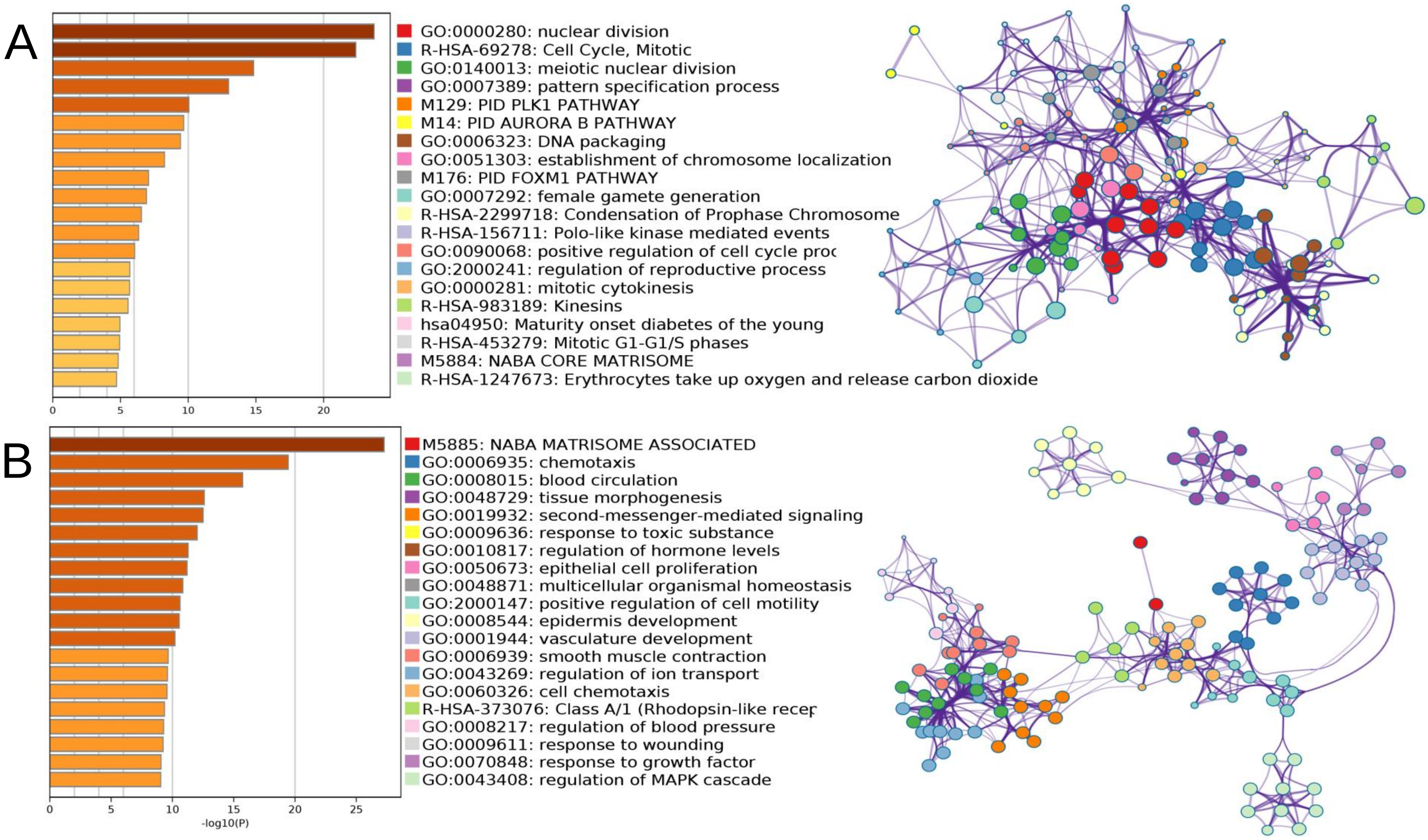

Table S1. The clinicopathological information of training and validation cohorts.

	TCGA[1] (n=415)	MSKCC[2] (n=138)	GSE116918[3] (n=223)
Age			
≤60	177	-	31
>60	238	-	192
Gleason score			
≤7	228	117	127
>7	187	21	96
Pathology T stage			
≤T2	143	131	127
>T2	272	7	96
Pathology N stage			
N0	338	-	-
N1	77	-	-
Recurrent			
Yes	71	35	51
No	344	103	172

1. The Cancer Genome Atlas (TCGA) : https://portal.gdc.cancer.gov/
2. MSKCC cohort/GSE21032: https://www.ncbi.nlm.nih.gov/geo/query/acc.cgi?acc=GSE21032
3. GSE116918: https://www.ncbi.nlm.nih.gov/geo/query/acc.cgi?acc=GSE116918

Table S2. The mRNA expression of KLF family members among normal and tumor tissues.

KLFs	Normal (FPKM, Mean ± SD)	Tumor (FPKM, Mean ± SD)	P value
KLF1	0.028±0.041	0.06±0.055	<0.001*
KLF2	14.363±10.961	13.517±8.027	0.446
KLF3	17.041±7.273	15.901±7.31	0.043*
KLF4	17.592±16.149	14.37±14.284	0.028*
KLF5	25.484±24.403	11.216±15.427	<0.001*
KLF6	23.584±19.86	17.813±21.992	0.002*
KLF7	3.131±1.525	2.195±0.93	<0.001*
KLF8	0.791±0.382	0.39±0.284	<0.001*
KLF9	27.46±11.407	21.268±9.114	<0.001*
KLF10	20.092±16.828	12.056±6.895	<0.001*
KLF11	5.873±2.204	3.897±1.691	<0.001*
KLF12	1.299±0.761	0.873±0.396	<0.001*
KLF13	6.583±2.848	5.571±3.171	0.001*
KLF14	0.038±0.034	0.036±0.019	0.875
KLF15	9.953±5.201	13.095±6.71	0.003*
KLF16	3.939±2.054	7.391±3.223	<0.001*
KLF17	0.046±0.062	0.024±0.03	<0.001*

*, P<0.05

Table S3. The promoter methylation of KLF family members among normal and tumor tissues.

KLFs	Normal (β value, Median, range)	Tumor (β value, Median, range)	P value
KLF1	0.792 (0.732-0.861)	0.817 (0.722-0.9)	<0.001*
KLF2	0.256 (0.236-0.269)	0.252 (0.228-0.275)	0.233
KLF3	0.045 (0.034-0.062)	0.062 (0.029-0.104)	<0.001*
KLF4	0.027 (0.022-0.039)	0.028 (0.016-0.042)	0.329
KLF5	0.037 (0.028-0.048)	0.035 (0.023-0.048)	0.002*
KLF6	0.107 (0.096-0.12)	0.112 (0.098-0.126)	<0.001*
KLF7	0.048 (0.04-0.063)	0.061 (0.028-0.133)	<0.001*
KLF8	0.317 (0.239-0.521)	0.711 (0.3-0.925)	<0.001*
KLF9	0.07 (0.05-0.101)	0.073 (0.036-0.133)	0.175
KLF10	0.039 (0.033-0.046)	0.037 (0.026-0.051)	0.002*
KLF11	0.292 (0.126-0.464)	0.38 (0.102-0.67)	<0.001*
KLF12	0.049 (0.036-0.061)	0.053 (0.036-0.173)	<0.001*
KLF13	0.106 (0.082-0.13)	0.108 (0.049-0.177)	<0.001*
KLF14	0.123 (0.086-0.154)	0.135 (0.057-0.312)	<0.001*
KLF15	0.041 (0.029-0.055)	0.034 (0.019-0.052)	<0.001*
KLF16	0.096 (0.056-0.12)	0.099 (0.057-0.146)	<0.001*
KLF17	0.931 (0.908-0.959)	0.892 (0.806-0.947)	<0.001*

*, P <0.05

The Beta value indicates level of DNA methylation ranging from 0 (unmethylated) to 1 (fully methylated).

Table S4. The coefficient of KLFs and clinical features conducted by the Cox proportional hazard regression analyses.

Characteristics	Co-ef	Exp(co-ef)	Se (co-ef)	z	Pr (>$\vert z \vert$)
Gleason score	0.542	1.719	0.139	3.912	<0.001[*]
Pathology T stage	0.559	1.749	0.268	2.084	0.038[*]
KLF5	-0.040	0.960	0.019	-2.088	0.038[*]
KLF13	0.077	1.080	0.036	2.127	0.033[*]

Co-ef, co-efficient; Exp (co-ef), Expectation (co-ef); Se (co-ef), standard error (co-ef); *, P < 0.05

Table S5. The distribution of TIICs among KLF-F and KLF-P group.

Immune cells	KLF-F (Mean ± SD)	KLF-P (Mean ± SD)	P value
B cells naive	0.053±0.037	0.051±0.037	0.518
Plasma cells	0.134±0.08	0.113±0.08	0.020*
T cells CD8	0.089±0.046	0.092±0.046	0.531
T cells CD4 memory resting	0.236±0.066	0.233±0.066	0.765
T cells follicular helper	0.034±0.027	0.031±0.027	0.227
T cells regulatory (Tregs)	0.015±0.021	0.019±0.021	0.070
NK cells activated	0.041±0.026	0.044±0.026	0.201
Monocytes	0.015±0.018	0.013±0.018	0.360
Macrophages M0	0.067±0.058	0.067±0.058	0.977
Macrophages M1	0.069±0.035	0.073±0.035	0.353
Macrophages M2	0.084±0.046	0.117±0.046	<0.001*
Dendritic cells resting	0.033±0.028	0.028±0.028	0.108
Dendritic cells activated	0.003±0.007	0.003±0.007	0.878
Mast cells resting	0.112±0.06	0.099±0.06	0.024*

* P<0.05

Table S6. The result of response to immunotherapy based on TIDE algorithm method.

Patient	Responde	TIDE	IFNG	MSI Score	CD274	CD8	CTL.flag	Dysfunction	Exclusion	MDSC	CAF	TAM M2
TCGA-J9-A8CK	FALSE	2.3	-0.76	0.31	-0.32	-0.32	FALSE	0.72	2.3	0.09	0.26	0
TCGA-VP-A87C	FALSE	2.19	2.24	0.17	0.86	1.74	TRUE	2.19	-1.37	-0.14	0.04	-0.1
TCGA-HI-7168	FALSE	2.15	-0.69	0.13	0.08	-0.72	FALSE	0.16	2.15	0.1	0.25	-0.01
TCGA-HC-7079	FALSE	2.13	2.62	0.11	2.46	1.44	TRUE	2.13	-2.3	-0.19	-0.01	-0.14
TCGA-J9-A8CM	FALSE	2.1	-0.32	0.32	-0.29	-0.41	FALSE	0.24	2.1	0.06	0.2	0.05
TCGA-YL-A8SJ	FALSE	2.09	-1.25	0.11	-0.47	-0.83	FALSE	0.02	2.09	0.08	0.2	0.04
TCGA-ZG-A9MC	FALSE	1.98	1.67	0.09	1.14	1.07	TRUE	1.98	-1.1	-0.1	0.06	-0.12
TCGA-J4-A6G1	FALSE	1.84	0.55	0.27	0.39	1.31	TRUE	1.84	-0.92	-0.08	-0.03	-0.03
TCGA-G9-6361	FALSE	1.84	-0.7	0.43	-0.17	-0.36	FALSE	-0.17	1.84	0.06	0.17	0.05
TCGA-G9-6365	FALSE	1.84	0.06	0.41	-0.15	0.25	FALSE	0.19	1.84	0.06	0.2	0.02
TCGA-KK-A7AV	FALSE	1.79	1.12	0.46	0.33	1.06	TRUE	1.79	-1.81	-0.12	-0.09	-0.06
TCGA-KK-A7B2	FALSE	1.77	1.14	0.49	0.2	0.98	TRUE	1.77	-0.37	-0.04	0.03	-0.05
TCGA-KK-A7B0	FALSE	1.7	0.11	0.73	0.18	0.74	TRUE	1.7	-0.27	-0.02	-0.05	0.02
TCGA-HC-8266	FALSE	1.66	2.24	0.19	1.24	2.18	TRUE	1.66	-1.94	-0.13	-0.04	-0.11
TCGA-G9-6379	FALSE	1.65	0.48	0.59	0.03	0.84	TRUE	1.65	-0.27	-0.01	-0.04	0
TCGA-KK-A8I4	FALSE	1.64	0.96	0.11	0.36	1.45	TRUE	1.64	-0.25	-0.06	0.09	-0.06
TCGA-ZG-A9L2	FALSE	1.62	0.3	0.31	-0.03	0.25	FALSE	0.88	1.62	0.02	0.23	0
TCGA-G9-6347	FALSE	1.57	0	0.76	0.23	0.85	TRUE	1.57	0.44	-0.02	0.09	0
TCGA-TK-A8OK	FALSE	1.56	-0.55	0.18	0.38	-0.6	FALSE	0.76	1.56	-0.02	0.3	-0.03
TCGA-ZG-A9NI	FALSE	1.53	-0.69	0.62	-0.2	-0.58	FALSE	0.31	1.53	0.05	0.16	0.03
TCGA-V1-A9Z8	FALSE	1.5	-0.56	0.39	-0.3	0.01	FALSE	0.44	1.5	0.04	0.16	0.03
TCGA-EJ-AB20	FALSE	1.48	-0.12	0.37	0.29	1.23	FALSE	1.17	1.48	0.02	0.2	0.01
TCGA-VN-A88I	FALSE	1.47	-1.08	0.18	-0.1	0.42	TRUE	1.47	1.57	-0.01	0.26	-0.01
TCGA-J9-A52E	FALSE	1.45	0.63	0.08	-0.18	1.48	FALSE	0.73	1.45	0.08	0.12	0.02
TCGA-V1-A8MU	FALSE	1.45	0.15	0.33	-0.03	0.88	TRUE	1.45	0.28	-0.04	0.11	-0.02
TCGA-ZG-A9LU	FALSE	1.44	0.61	0.41	0.38	0.47	TRUE	1.44	-0.6	0.02	-0.06	-0.06
TCGA-XJ-A9DI	FALSE	1.43	0.89	0.17	0.54	1.29	TRUE	1.43	-0.36	-0.06	0.07	-0.07
TCGA-J9-A52C	FALSE	1.41	0.45	0.4	0.24	0.95	TRUE	1.41	0.68	-0.03	0.22	-0.08
TCGA-ZG-A8QY	FALSE	1.41	-0.39	0.69	0.34	-0.98	FALSE	0.09	1.41	0.08	0.1	0.03
TCGA-HC-A6AS	FALSE	1.41	0.2	0.82	0.15	0.46	TRUE	1.41	0.04	-0.03	0.06	-0.03
TCGA-ZG-A9L4	FALSE	1.41	0.02	0.32	-0.26	-0.08	FALSE	0.29	1.41	0.05	0.21	-0.04
TCGA-J4-A67M	FALSE	1.4	-0.31	0.73	-0.04	0.78	TRUE	1.4	0.24	-0.02	0.04	0.01
TCGA-QU-A6IM	FALSE	1.4	0.74	0.86	1.04	1.75	TRUE	1.4	-0.64	0	-0.1	0
TCGA-HC-7745	FALSE	1.38	0.85	0.19	0.25	0.61	TRUE	1.38	-0.26	-0.1	0.12	-0.05
TCGA-ZG-A9L9	FALSE	1.37	1.04	0.42	0.13	0.54	FALSE	0.6	1.37	0.04	0.17	-0.01
TCGA-HC-A6HX	FALSE	1.36	-0.86	0.71	0.37	0.29	TRUE	1.36	0.35	0.04	-0.01	0.02
TCGA-EJ-5525	FALSE	1.35	-0.35	0.11	-0.17	-1.02	FALSE	-1.18	1.35	0.06	0.11	0.04
TCGA-V1-A9Z7	FALSE	1.34	0.17	0.4	0.19	-0.1	FALSE	0.02	1.34	0.03	0.12	0.05
TCGA-FC-7961	FALSE	1.34	-0.35	0.37	0.08	-0.05	FALSE	-0.76	1.34	0.04	0.14	0.01
TCGA-HC-8264	FALSE	1.31	0.1	0.4	0.47	-0.64	FALSE	-0.22	1.31	-0.03	0.22	0.01
TCGA-V1-A9OX	FALSE	1.3	-0.3	0.57	-0.05	0.56	TRUE	1.3	-0.59	-0.03	-0.06	-0.01
TCGA-KC-A7FE	FALSE	1.3	-0.58	0.4	0.06	-0.56	FALSE	0.78	1.3	-0.01	0.2	0.01
TCGA-QU-A6IO	FALSE	1.3	-0.39	0.86	0.23	0.62	TRUE	1.3	-0.55	0	-0.12	0.03
TCGA-V1-A9O9	FALSE	1.28	1.99	0.25	0.03	1.12	TRUE	1.28	-1.73	-0.08	-0.11	-0.07
TCGA-G9-7523	FALSE	1.27	0.36	0.29	0.4	0.65	FALSE	0.95	1.27	-0.05	0.27	-0.02
TCGA-HC-7742	FALSE	1.26	-0.12	0.31	-0.31	-0.28	FALSE	-0.43	1.26	0.05	0.14	0.01
TCGA-XQ-A8TB	FALSE	1.25	-0.17	0.28	-0.24	-0.15	FALSE	-0.21	1.25	0.03	0.17	-0.01
TCGA-HI-7170	FALSE	1.24	0.22	0.35	0.15	0.28	TRUE	1.24	0.86	0	0.22	-0.08
TCGA-ZG-A9LN	FALSE	1.24	2.47	0.46	0.7	2.21	TRUE	1.24	-0.5	-0.05	0.05	-0.08
TCGA-ZG-A9L1	FALSE	1.23	-1.26	0.45	-0.68	-0.74	FALSE	-0.41	1.23	0.07	0.11	0.01
TCGA-YL-A8SB	FALSE	1.22	-1	0.38	-0.31	-0.27	FALSE	0.38	1.22	0	0.15	0.04
TCGA-EJ-7328	FALSE	1.22	0.83	0.21	0.2	0.81	TRUE	1.22	-0.58	-0.07	0.04	-0.05
TCGA-YL-A9WX	FALSE	1.21	-0.3	0.24	0	0.48	TRUE	1.21	-0.67	-0.08	0.09	-0.1
TCGA-HI-7169	FALSE	1.21	-1.12	0.5	0.24	0.31	TRUE	1.21	0.65	-0.01	0.11	0
TCGA-YL-A8SK	FALSE	1.2	0.29	0.32	0.17	0.78	TRUE	1.2	-0.05	-0.01	0.08	-0.07
TCGA-VP-A879	FALSE	1.19	-1.23	0.37	-0.56	-0.09	FALSE	0.71	1.19	0	0.15	0.03
TCGA-VP-A87E	FALSE	1.19	0.98	0.57	0.33	1.04	TRUE	1.19	-0.81	-0.09	0.09	-0.11
TCGA-4L-AA1F	FALSE	1.17	1.58	0.03	0.36	0.29	TRUE	1.17	0.8	-0.08	0.29	-0.08
TCGA-VN-A88L	FALSE	1.16	-0.41	0.35	-0.27	-0.19	FALSE	-0.12	1.16	0.05	0.13	0
TCGA-V1-A9OH	FALSE	1.12	-0.13	0.59	-0.16	-0.08	FALSE	-0.27	1.12	0.06	0.06	0.05
TCGA-HC-A9TE	FALSE	1.12	0.91	0.09	0.31	0.46	TRUE	1.12	-0.94	-0.1	-0.02	-0.03
TCGA-EJ-8474	FALSE	1.12	-0.4	0.57	-0.23	-0.46	FALSE	-1.13	1.12	0.05	0.08	0.04
TCGA-G9-6356	FALSE	1.12	0.97	0.28	0.15	1.04	TRUE	1.12	0.3	-0.01	0.11	-0.04
TCGA-HC-7750	FALSE	1.11	-0.97	0.56	-0.23	-0.43	FALSE	0.5	1.11	0.01	0.14	0.03
TCGA-2A-A8VV	FALSE	1.1	-0.9	0.54	-0.37	-0.45	FALSE	-0.96	1.1	0.09	-0.01	0.09
TCGA-YL-A8HJ	FALSE	1.09	0.32	0.21	0.18	0.55	TRUE	1.09	0.6	-0.06	0.2	-0.03
TCGA-KK-A8I7	FALSE	1.09	0.25	0.26	-0.03	0.08	TRUE	1.09	-0.24	-0.06	0.04	-0.01

Patient	Responde	TIDE	IFNG	MSI Score	CD274	CD8	CTL.flag	Dysfunction	Exclusion	MDSC	CAF	TAM M2
TCGA-J4-A83K	FALSE	1.08	-0.87	0.48	-0.32	-0.43	FALSE	-0.01	1.08	0.03	0.09	0.05
TCGA-V1-A9OA	FALSE	1.07	0.06	0.23	-0.04	0.02	FALSE	0.76	1.07	0.02	0.14	0
TCGA-KK-A7B3	FALSE	1.06	0.49	0.2	-0.03	0.49	TRUE	1.06	0.09	-0.04	0.04	0.01
TCGA-KK-A7AW	FALSE	1.06	1	0.84	0.37	0.92	TRUE	1.06	-0.68	0.01	-0.14	0.02
TCGA-EJ-7791	FALSE	1.06	-0.21	0.28	0	-0.21	FALSE	0.75	1.06	-0.04	0.24	-0.03
TCGA-G9-6498	FALSE	1.05	0.02	0.36	0.94	1.14	TRUE	1.05	-0.38	-0.03	0.04	-0.06
TCGA-H9-A6BY	FALSE	1.04	-1.13	0.91	-0.13	0.01	FALSE	-0.17	1.04	0.12	-0.09	0.12
TCGA-KC-A4BL	FALSE	1.04	0.04	0.31	0.06	0.15	TRUE	1.04	0.05	-0.01	0.09	-0.06
TCGA-KK-A7B1	FALSE	1.03	-0.8	0.26	-0.3	-0.47	FALSE	0.15	1.03	0.05	0.11	0
TCGA-M7-A723	FALSE	1.02	1.77	0.69	0.5	1.19	TRUE	1.02	-0.08	-0.07	0.08	-0.03
TCGA-G9-6364	FALSE	1.02	0.37	0.27	-0.23	0.24	FALSE	-0.08	1.02	-0.03	0.19	0.01
TCGA-H9-A6BX	FALSE	1.02	0.04	0.27	0.08	-0.15	FALSE	1.33	1.02	-0.03	0.22	-0.02
TCGA-V1-A9Z9	FALSE	1.02	-0.27	0.49	-0.02	-0.39	FALSE	0.31	1.02	0.02	0.1	0.03
TCGA-KK-A6E6	FALSE	1.02	0.46	0.49	-0.27	0.48	FALSE	-0.35	1.02	0.02	0.15	-0.02
TCGA-G9-6378	FALSE	1.01	0.07	0.52	0.12	-0.26	FALSE	0.37	1.01	-0.01	0.16	0.01
TCGA-KK-A6E3	FALSE	1	0.69	0.86	0.24	0.33	TRUE	1	0.23	-0.02	0.04	0.01
TCGA-V1-A8ML	FALSE	0.99	-0.27	0.71	0.31	-0.19	FALSE	-0.2	0.99	0.05	0.07	0.03
TCGA-HC-7737	FALSE	0.98	0.34	0.37	0.11	0.73	TRUE	0.98	-0.78	-0.1	0.09	-0.1
TCGA-HC-A76W	FALSE	0.97	-0.2	0.2	0.07	-0.6	FALSE	0.57	0.97	0.02	0.16	-0.02
TCGA-EJ-AB27	FALSE	0.97	0.64	0.67	0.06	0.29	TRUE	0.97	-0.95	-0.05	-0.05	-0.04
TCGA-YL-A9WY	FALSE	0.96	1.25	0.14	0.08	0.58	TRUE	0.96	-0.14	-0.05	0.09	-0.05
TCGA-CH-5754	FALSE	0.96	2.67	0.15	0.7	1.11	TRUE	0.96	-1.06	0.02	-0.09	-0.09
TCGA-G9-7525	FALSE	0.96	-0.83	0.71	-0.12	-0.31	FALSE	-0.03	0.96	0.02	0.07	0.06
TCGA-KC-A4BR	FALSE	0.96	-0.55	0.4	0.8	-0.4	FALSE	0.99	0.96	0.01	0.22	-0.08
TCGA-V1-A8MF	FALSE	0.95	0.53	0.7	0.27	0.88	TRUE	0.95	-1.45	-0.09	-0.09	-0.03
TCGA-CH-5745	FALSE	0.94	0.5	0.39	0.26	0.36	TRUE	0.94	-0.69	-0.11	0.1	-0.09
TCGA-J4-8198	FALSE	0.93	-0.43	0.33	-0.01	-0.58	FALSE	-0.67	0.93	0	0.09	0.05
TCGA-XK-AAJU	FALSE	0.93	-0.44	0.48	0.02	0.53	TRUE	0.93	-0.47	-0.03	-0.02	-0.02
TCGA-J4-A67S	FALSE	0.93	0.07	0.8	0.01	0.75	TRUE	0.93	-0.42	0.03	-0.12	0.01
TCGA-KK-A8IL	FALSE	0.92	1.65	0.38	0.54	1.06	TRUE	0.92	-1.34	-0.05	-0.01	-0.13
TCGA-FC-A8O0	FALSE	0.92	-1.11	0.51	0.63	-1.23	FALSE	0.33	0.92	0.03	0.07	0.04
TCGA-HC-A6AP	FALSE	0.92	-0.3	0.79	0.15	0.2	TRUE	0.92	0.26	0.01	-0.02	0.04
TCGA-G9-6353	FALSE	0.91	-1.16	0.56	-0.36	-0.14	FALSE	0.9	0.91	0	0.1	0.03
TCGA-V1-A9ZG	FALSE	0.91	-0.58	0.28	-0.03	-0.62	FALSE	0.3	0.91	0.02	0.12	0
TCGA-EJ-7788	FALSE	0.9	-0.18	0.34	-0.3	-0.33	FALSE	-0.8	0.9	0	0.1	0.04
TCGA-EJ-5504	FALSE	0.89	0.31	0.45	0.03	-0.42	FALSE	-0.83	0.89	-0.01	0.14	0.01
TCGA-G9-6329	FALSE	0.89	0.41	0.39	0.01	0.75	TRUE	0.89	0.75	0.03	0.08	0
TCGA-EJ-A8FU	FALSE	0.88	0.16	0.41	0.03	0.05	FALSE	1.13	0.88	-0.02	0.2	-0.04
TCGA-J4-A6M7	FALSE	0.86	-1.09	0.52	-0.33	-0.51	FALSE	-1.07	0.86	0.05	0.02	0.06
TCGA-TP-A8TT	FALSE	0.85	2.74	0.41	0.63	1.68	TRUE	0.85	-1.37	-0.13	0.01	-0.09
TCGA-EJ-7327	FALSE	0.85	0.27	0.17	-0.33	-0.23	FALSE	0.11	0.85	0	0.14	0
TCGA-KC-A4BV	FALSE	0.83	1.28	0.33	-0.09	0.61	FALSE	0.36	0.83	0	0.12	0
TCGA-EJ-7315	FALSE	0.82	0.68	0.18	-0.23	1.03	TRUE	0.82	-0.18	-0.06	0.06	-0.02
TCGA-G9-6385	FALSE	0.82	-0.48	0.56	-0.09	-0.2	FALSE	0.5	0.82	0	0.13	0
TCGA-J9-A8CL	FALSE	0.82	0.67	0.35	0.08	-0.27	FALSE	0.37	0.82	0	0.14	-0.01
TCGA-KK-A59V	FALSE	0.82	2.63	0.69	0.85	3.15	TRUE	0.82	-1.83	-0.03	-0.2	-0.06
TCGA-J4-A67N	FALSE	0.81	0.17	0.73	0.99	1.18	TRUE	0.81	-0.61	0	-0.11	0.02
TCGA-CH-5743	FALSE	0.81	0.51	0.46	0.51	1.39	TRUE	0.81	-1.33	-0.08	0	-0.11
TCGA-V1-A9OT	FALSE	0.8	-1	0.3	-0.1	1.51	TRUE	0.8	-0.11	0.02	-0.08	0.04
TCGA-J4-A6G3	FALSE	0.8	1.01	0.25	0.33	0.93	TRUE	0.8	-0.76	0	0	-0.1
TCGA-X4-A8KS	FALSE	0.8	-0.49	0.53	0.17	0.63	TRUE	0.8	-0.53	-0.02	-0.08	0.01
TCGA-YL-A8SO	FALSE	0.79	-0.96	0.34	-0.31	-0.51	FALSE	0.21	0.79	0.07	0.01	0.04
TCGA-EJ-A7NM	FALSE	0.79	1.07	0.26	-0.34	1.05	TRUE	0.79	0.03	0.03	-0.05	0.02
TCGA-HC-7747	FALSE	0.77	1.28	0.62	0.41	0.79	TRUE	0.77	-0.78	-0.06	-0.02	-0.04
TCGA-EJ-7318	FALSE	0.77	0.96	0.76	-0.02	0.08	TRUE	0.77	0.14	0.03	-0.04	0.02
TCGA-EJ-5501	FALSE	0.76	-0.06	0.37	0.02	0.04	FALSE	0.38	0.76	0.06	0.06	-0.01
TCGA-HC-7210	FALSE	0.76	0.76	0.32	0.75	0.48	TRUE	0.76	-0.45	-0.12	0.14	-0.08
TCGA-EJ-A7NG	FALSE	0.76	-0.89	0.4	-0.27	-0.54	FALSE	0.75	0.76	-0.02	0.18	-0.04
TCGA-G9-7509	FALSE	0.75	-0.73	0.55	-0.22	-0.24	FALSE	-0.55	0.75	0.01	0.06	0.04
TCGA-J4-A67Q	FALSE	0.72	-0.39	0.62	-0.06	-0.15	FALSE	1.28	0.72	0	0.09	0.01
TCGA-CH-5769	FALSE	0.72	0.51	0.54	-0.13	-0.24	FALSE	-0.86	0.72	0.11	0.04	-0.04
TCGA-XK-AAIV	FALSE	0.71	1.54	0.22	0.66	1.26	TRUE	0.71	-0.93	-0.05	-0.03	-0.06
TCGA-EJ-A7NJ	FALSE	0.68	0.83	0.72	0.08	0.69	TRUE	0.68	-1.29	-0.08	-0.07	-0.04
TCGA-ZG-A9L6	FALSE	0.68	-0.35	0.23	0.06	-0.46	FALSE	1.08	0.68	-0.07	0.17	0
TCGA-YL-A8SP	FALSE	0.67	-1.19	0.32	-0.28	-0.44	FALSE	-0.86	0.67	0.09	-0.05	0.06
TCGA-KK-A8IG	FALSE	0.67	-0.13	0.43	-0.01	0.1	FALSE	0.37	0.67	-0.03	0.14	-0.01
TCGA-2A-AAYU	FALSE	0.67	-0.67	0.51	-0.53	-0.23	FALSE	-0.74	0.67	0.03	0.02	0.04
TCGA-ZG-A8QZ	FALSE	0.67	0.5	0.41	0.06	0.06	FALSE	0.71	0.67	-0.01	0.15	-0.04
TCGA-FC-7708	FALSE	0.66	0.29	0.24	-0.13	0.41	TRUE	0.66	0.67	-0.06	0.25	-0.07
TCGA-V1-A8X3	FALSE	0.66	0.42	0.54	-0.07	-0.23	FALSE	-0.02	0.66	0	0.12	-0.01

Patient	Responde	TIDE	IFNG	MSI Score	CD274	CD8	CTL.flag	Dysfunction	Exclusion	MDSC	CAF	TAM M2
TCGA-ZG-A8QW	FALSE	0.66	0.94	0.49	0.29	0.5	TRUE	0.66	-0.11	0.02	0.01	-0.04
TCGA-V1-A9ZR	FALSE	0.65	0.14	0.32	-0.05	0.26	FALSE	1.19	0.65	-0.06	0.15	0.01
TCGA-ZG-A9M4	FALSE	0.65	1.32	0.33	0.81	1.55	TRUE	0.65	-1.25	-0.05	-0.11	-0.02
TCGA-XK-AAJ3	FALSE	0.64	-0.5	0.7	0.32	-0.01	FALSE	0.09	0.64	0.04	-0.01	0.05
TCGA-M7-A71Y	FALSE	0.63	-0.53	0.65	0.05	0.34	FALSE	0.63	0.63	0.01	0.05	0.03
TCGA-KK-A8IC	FALSE	0.62	-0.11	0.23	-0.33	-0.17	FALSE	0.58	0.62	0.01	0.16	-0.07
TCGA-J4-A67L	FALSE	0.62	1.06	0.84	-0.01	0.96	TRUE	0.62	-0.29	-0.01	-0.04	0
TCGA-V1-A8MG	FALSE	0.61	0.42	0.36	-0.02	0.47	TRUE	0.61	0.55	0.04	0.03	0
TCGA-EJ-5531	FALSE	0.61	-0.09	0.46	0.13	0.88	TRUE	0.61	-0.68	-0.09	0.04	-0.04
TCGA-G9-6363	FALSE	0.61	0.67	0.86	-0.19	-0.02	FALSE	-1.66	0.61	0.05	0.01	0.02
TCGA-2A-A8VX	FALSE	0.61	-1.23	0.82	-0.26	-0.39	FALSE	-0.99	0.61	0.09	-0.06	0.06
TCGA-XJ-A9DQ	FALSE	0.61	-1.2	0.48	-0.08	0.06	FALSE	0.27	0.61	0.02	-0.01	0.06
TCGA-V1-A9OY	FALSE	0.61	0.14	0.4	-0.23	0.62	FALSE	0.79	0.61	0.03	0.04	0.02
TCGA-Y6-A8TL	FALSE	0.6	-0.76	0.7	0	-0.26	FALSE	-0.25	0.6	0.04	0.03	0.01
TCGA-EJ-8472	FALSE	0.6	-0.58	0.55	-0.29	-0.47	FALSE	-0.86	0.6	0.07	0.02	0
TCGA-J4-A83L	FALSE	0.6	-0.47	0.62	0.27	-0.9	FALSE	-0.07	0.6	-0.02	0.17	-0.04
TCGA-EJ-7115	FALSE	0.59	-0.74	0.39	-0.15	-0.23	FALSE	0.66	0.59	-0.01	0.08	0.03
TCGA-H9-7775	FALSE	0.58	-0.74	0.44	-0.36	-0.67	FALSE	-0.68	0.58	-0.03	0.09	0.04
TCGA-HC-A8D1	FALSE	0.58	0.97	0.52	0.17	0.47	TRUE	0.58	-0.11	-0.06	0.1	-0.05
TCGA-XK-AAJR	FALSE	0.58	-1.06	0.39	-0.18	-0.74	FALSE	-0.04	0.58	0.03	-0.01	0.07
TCGA-YL-A9WK	FALSE	0.57	0.12	0.34	0.25	0.33	FALSE	0.25	0.57	-0.01	0.08	0.01
TCGA-V1-A8MM	FALSE	0.57	0.97	0.47	0.1	0.26	TRUE	0.57	-0.19	-0.01	-0.04	0.01
TCGA-YL-A8SI	FALSE	0.56	-0.3	0.62	0.07	-0.02	FALSE	0.43	0.56	-0.03	0.14	-0.02
TCGA-SU-A7E7	FALSE	0.56	1.07	0.35	0.28	0.28	TRUE	0.56	-0.7	-0.02	0.03	-0.12
TCGA-YL-A8S8	FALSE	0.56	-0.19	0.27	-0.06	-0.39	FALSE	0.02	0.56	0.07	0.02	-0.01
TCGA-YL-A9WH	FALSE	0.55	0.33	0.54	-0.28	-0.92	FALSE	-0.58	0.55	0.12	-0.04	0
TCGA-KK-A8I5	FALSE	0.53	-0.35	0.46	-0.45	-0.69	FALSE	-0.46	0.53	0.01	0.1	-0.02
TCGA-XJ-A83F	FALSE	0.53	-1.06	0.55	-0.46	-0.65	FALSE	-0.34	0.53	0.06	-0.04	0.06
TCGA-V1-A9O7	FALSE	0.52	-0.05	0.21	0.07	0.15	FALSE	0.7	0.52	0	0.14	-0.05
TCGA-J4-A67O	FALSE	0.51	-0.14	0.89	0.39	-0.15	FALSE	0.97	0.51	0.01	0.05	0.01
TCGA-HC-7817	FALSE	0.5	0.11	0.42	0.28	0.4	TRUE	0.5	0.32	-0.03	0.16	-0.07
TCGA-VN-A88K	FALSE	0.5	-0.35	0.34	0.02	-0.3	FALSE	-0.28	0.5	0.05	0.02	0
TCGA-HC-A8D0	FALSE	0.49	-0.4	0.37	-0.03	0.49	TRUE	0.49	0.74	0.02	0.06	0.03
TCGA-J4-A67K	FALSE	0.49	0.64	0.85	0.15	0.84	TRUE	0.49	-0.27	0.04	-0.12	0.03
TCGA-J9-A52B	FALSE	0.49	0.91	0.38	-0.16	1.23	FALSE	0.94	0.49	0.07	-0.01	0.01
TCGA-G9-6494	FALSE	0.48	-0.39	0.57	-0.1	-0.65	FALSE	-0.45	0.48	-0.02	0.06	0.04
TCGA-ZG-A9L0	FALSE	0.48	0.75	0.27	0.31	0.34	TRUE	0.48	0.07	-0.01	0.09	-0.07
TCGA-HC-7081	FALSE	0.48	0.18	0.09	-0.29	0.41	FALSE	0.8	0.48	-0.1	0.19	0
TCGA-XK-AAJT	FALSE	0.48	0.43	0.44	0.3	-0.03	FALSE	1.27	0.48	-0.01	0.11	-0.03
TCGA-KK-A5A1	FALSE	0.47	0.27	0.62	0.42	-0.24	FALSE	-1	0.47	0.03	-0.03	0.05
TCGA-EJ-5542	FALSE	0.47	0.02	0.4	-0.16	-0.27	FALSE	-1.1	0.47	-0.03	0.08	0.03
TCGA-YL-A8HK	FALSE	0.47	0.32	0.1	-0.34	0.15	FALSE	0.58	0.47	0.01	0.11	-0.04
TCGA-HC-A631	FALSE	0.47	1.03	0.74	-0.41	-0.57	FALSE	-1.23	0.47	0.1	-0.05	0.01
TCGA-EJ-A65B	FALSE	0.46	-0.95	0.74	-0.17	-0.42	FALSE	0.7	0.46	0.01	0.02	0.04
TCGA-V1-A9ZI	FALSE	0.45	-0.25	0.14	-0.35	-0.22	FALSE	0.46	0.45	0.01	0.01	0.05
TCGA-HC-A4ZV	FALSE	0.45	0.37	0.23	0.06	0.57	FALSE	0.08	0.45	0.04	0.01	0.01
TCGA-XJ-A9DK	FALSE	0.44	-0.9	0.49	0.01	0.42	FALSE	0.36	0.44	0	0.06	0.01
TCGA-EJ-A8FO	FALSE	0.43	-0.62	0.51	-0.21	-0.36	FALSE	-0.09	0.43	0	0.03	0.04
TCGA-ZG-A9L5	FALSE	0.43	-0.62	0.54	0.24	-0.68	FALSE	-0.78	0.43	0.07	-0.04	0.03
TCGA-V1-A8MK	FALSE	0.43	0.37	0.57	0.09	0.1	FALSE	0.34	0.43	-0.01	0.08	0
TCGA-EJ-A46D	FALSE	0.42	-0.94	0.57	-0.4	0.17	FALSE	0.2	0.42	0	0	0.05
TCGA-G9-6367	FALSE	0.42	0.14	0.67	0.24	0.04	FALSE	0.65	0.42	-0.03	0.12	-0.02
TCGA-CH-5763	FALSE	0.4	-0.64	0.48	0.37	0.16	FALSE	0.21	0.4	-0.04	0.19	-0.08
TCGA-X4-A8KQ	FALSE	0.4	-0.06	0.4	-0.3	0.1	FALSE	0.75	0.4	0.01	0.09	-0.04
TCGA-YL-A8SC	FALSE	0.39	1.3	0.42	0.69	0.99	TRUE	0.39	-1.03	-0.02	-0.07	-0.07
TCGA-J9-A8CP	FALSE	0.38	-0.58	0.37	0.91	-0.76	FALSE	0.46	0.38	0.01	0.05	0
TCGA-KK-A8IM	FALSE	0.37	0.27	0.28	-0.2	0.36	TRUE	0.37	0.15	-0.02	0.06	-0.02
TCGA-HC-A6AQ	FALSE	0.37	-0.58	0.85	-0.16	0.3	TRUE	0.37	0.11	0.04	-0.11	0.07
TCGA-ZG-A9LB	FALSE	0.37	0.37	0.71	2.76	0.79	TRUE	0.37	0.19	0.06	-0.01	-0.03
TCGA-HC-7232	FALSE	0.36	-0.12	0.36	-0.37	-0.24	FALSE	-0.38	0.36	-0.01	0.04	0.03
TCGA-CH-5752	FALSE	0.35	-0.51	0.46	-0.45	-0.46	FALSE	-1.13	0.35	0.06	-0.04	0.03
TCGA-G9-6496	FALSE	0.35	-0.27	0.35	-0.09	-0.41	FALSE	0.16	0.35	0.06	-0.04	0.03
TCGA-EJ-7317	FALSE	0.34	-0.97	0.77	0.05	-0.7	FALSE	-0.22	0.34	0.01	-0.02	0.06
TCGA-HC-A48F	FALSE	0.33	-1.12	0.37	-0.6	-0.82	FALSE	-0.99	0.33	0.04	-0.03	0.04
TCGA-HI-7171	FALSE	0.32	-0.08	0.47	-0.52	0.43	FALSE	-0.99	0.32	0.07	-0.08	0.05
TCGA-G9-6351	FALSE	0.32	-0.86	0.64	-0.18	0.32	FALSE	-0.36	0.32	0	0.03	0.01
TCGA-EJ-5498	FALSE	0.31	0.41	0.35	0.19	0.47	FALSE	0.21	0.31	-0.06	0.16	-0.04
TCGA-CH-5761	FALSE	0.31	-0.44	0.18	-0.06	-0.93	FALSE	-0.79	0.31	0.08	-0.02	-0.02
TCGA-2A-A8W3	FALSE	0.31	-0.18	0.47	-0.14	-0.16	FALSE	-0.59	0.31	0.05	-0.03	0.03
TCGA-QU-A6IP	FALSE	0.31	-0.72	0.71	-0.32	-0.07	FALSE	0.41	0.31	0.04	-0.03	0.02

Patient	Responde	TIDE	IFNG	MSI Score	CD274	CD8	CTL.flag	Dysfunction	Exclusion	MDSC	CAF	TAM M2
TCGA-EJ-A65D	FALSE	0.3	-1.43	0.9	-0.24	0.12	FALSE	-0.59	0.3	0.13	-0.2	0.1
TCGA-HC-A9TH	FALSE	0.29	1.75	0.35	0.08	-0.41	FALSE	-0.26	0.29	0.05	0.06	-0.06
TCGA-EJ-7792	FALSE	0.29	-0.01	0.23	0.06	-0.26	FALSE	0.78	0.29	-0.09	0.19	-0.04
TCGA-VP-AA1N	FALSE	0.28	0.58	0.6	0.02	0.36	TRUE	0.28	-0.1	-0.03	0.05	-0.04
TCGA-XK-AAIR	FALSE	0.28	-0.22	0.45	-0.13	0.4	TRUE	0.28	-0.22	-0.08	0.04	0.02
TCGA-ZG-A9LS	FALSE	0.28	1.02	0.23	0.14	0.73	FALSE	1.43	0.28	-0.07	0.16	-0.04
TCGA-HC-7233	FALSE	0.27	2.14	0.56	0.43	0.72	TRUE	0.27	-1.24	-0.13	0.06	-0.1
TCGA-G9-6377	FALSE	0.27	0.71	0.51	0.32	0.55	TRUE	0.27	-0.38	0.01	0.01	-0.07
TCGA-EJ-8470	FALSE	0.27	2.37	0.48	1.13	0.97	TRUE	0.27	-2.51	-0.13	-0.13	-0.12
TCGA-G9-7510	FALSE	0.26	0.34	0.43	0.09	-0.34	FALSE	1.1	0.26	-0.04	0.17	-0.07
TCGA-G9-6373	FALSE	0.25	0.49	0.5	0.08	0.22	TRUE	0.25	0.66	0.03	0.07	-0.01
TCGA-CH-5751	FALSE	0.23	2.13	0.16	0.99	2.24	TRUE	0.23	-1.43	-0.04	-0.13	-0.05
TCGA-G9-6370	FALSE	0.23	-0.37	0.48	0.37	0.48	FALSE	0.88	0.23	-0.04	0.12	-0.04
TCGA-KK-A8I8	FALSE	0.22	0.67	0.57	-0.43	0.23	FALSE	-1.08	0.22	0.08	-0.13	0.07
TCGA-YL-A8SL	FALSE	0.22	-0.88	0.46	-0.65	-0.96	FALSE	-0.92	0.22	0.13	-0.16	0.05
TCGA-KK-A7AQ	FALSE	0.22	-0.03	0.54	-0.04	-0.17	FALSE	1.11	0.22	0.04	-0.05	0.04
TCGA-EJ-5518	FALSE	0.22	0.14	0.46	-0.31	-0.49	FALSE	0.06	0.22	-0.02	0.08	-0.02
TCGA-G9-7519	FALSE	0.21	-0.56	0.87	0	0.04	FALSE	0.13	0.21	-0.03	0.07	0
TCGA-EJ-5512	FALSE	0.21	0.09	0.66	0.09	0.01	FALSE	-0.81	0.21	0.01	0.02	0
TCGA-CH-5771	FALSE	0.2	0.18	0.22	0.08	-0.01	FALSE	1.85	0.2	-0.1	0.22	-0.07
TCGA-HC-8261	FALSE	0.2	0.63	0.6	0.42	0.35	TRUE	0.2	-1.13	-0.04	-0.14	0
TCGA-EJ-A7NH	FALSE	0.2	-0.49	0.37	-0.12	0.24	FALSE	0.89	0.2	-0.01	-0.02	0.06
TCGA-ZG-A9LM	FALSE	0.19	0.4	0.39	0	-0.12	FALSE	-0.02	0.19	-0.02	0.06	-0.01
TCGA-KK-A59X	FALSE	0.19	0.07	0.85	0.54	-0.91	FALSE	-0.76	0.19	0.04	0	-0.02
TCGA-J4-A67R	FALSE	0.19	-0.05	0.66	-0.03	0.04	FALSE	1.49	0.19	0.01	0.02	0
TCGA-HC-A76X	FALSE	0.19	-1.3	0.73	-0.3	-0.15	FALSE	-1.39	0.19	0.08	-0.14	0.08
TCGA-EJ-5495	FALSE	0.19	1.23	0.16	0.27	0.59	FALSE	0.24	0.19	-0.05	0.13	-0.03
TCGA-G9-7522	FALSE	0.19	0.16	0.48	-0.16	0.32	TRUE	0.19	-0.07	-0.09	0.11	-0.03
TCGA-YL-A9WL	FALSE	0.18	-0.05	0.39	-0.5	-0.16	FALSE	-0.51	0.18	0.09	-0.12	0.05
TCGA-VP-A872	FALSE	0.18	-0.27	0.22	-0.19	0	FALSE	1.18	0.18	-0.01	0.07	-0.03
TCGA-G9-6348	FALSE	0.18	0.57	0.59	-0.02	0.71	TRUE	0.18	0.7	0.01	0.15	-0.05
TCGA-V1-A9ZK	FALSE	0.18	-0.49	0.51	-0.04	-0.21	FALSE	-0.04	0.18	-0.02	0.03	0.02
TCGA-HC-A632	FALSE	0.17	0.62	0.29	0.06	0.34	TRUE	0.17	-0.06	-0.04	-0.01	0.03
TCGA-KK-A7AZ	FALSE	0.17	-0.31	0.42	-0.39	-0.01	FALSE	-0.31	0.17	0.07	-0.11	0.06
TCGA-XQ-A8TA	FALSE	0.17	-1.66	0.24	-0.41	-0.84	FALSE	-0.93	0.17	0.14	-0.19	0.05
TCGA-EJ-A46B	FALSE	0.17	-1.03	0.7	-0.24	-0.47	FALSE	0.25	0.17	-0.03	0	0.05
TCGA-KC-A7F3	FALSE	0.16	-0.72	0.7	-0.01	0.08	FALSE	-0.39	0.16	0.06	-0.03	-0.01
TCGA-G9-A9S4	FALSE	0.16	-0.84	0.52	-0.3	-0.37	FALSE	-0.38	0.16	0.1	-0.14	0.06
TCGA-EJ-5524	FALSE	0.15	1.04	0.34	0.08	0.48	FALSE	-0.52	0.15	-0.05	0.11	-0.02
TCGA-EJ-5530	FALSE	0.15	-0.48	0.31	-0.35	-0.44	FALSE	-0.47	0.15	-0.03	0.03	0.02
TCGA-EJ-8469	FALSE	0.14	0.32	0.51	-0.05	-0.29	FALSE	-1.39	0.14	0.08	-0.09	0.03
TCGA-EJ-7793	FALSE	0.14	-0.62	0.69	-0.12	0.01	FALSE	-0.49	0.14	-0.01	-0.01	0.04
TCGA-ZG-A9KY	FALSE	0.13	1.67	0.72	1.47	2.38	TRUE	0.13	-1.2	0	-0.18	-0.01
TCGA-KK-A7B4	FALSE	0.13	0.67	0.31	-0.38	0.45	FALSE	-0.04	0.13	0.07	-0.09	0.03
TCGA-HC-A6AN	FALSE	0.13	-0.5	0.83	-0.16	-0.41	FALSE	0.2	0.13	0.02	-0.08	0.07
TCGA-KK-A59Z	FALSE	0.13	-0.13	0.21	0.39	-0.23	FALSE	0.81	0.13	-0.02	0.08	-0.03
TCGA-EJ-A46I	FALSE	0.12	-0.33	0.45	0	-0.08	FALSE	0.91	0.12	-0.02	0.04	0
TCGA-EJ-5511	FALSE	0.12	-0.47	0.29	-0.2	-0.77	FALSE	-0.56	0.12	-0.02	0.03	0.02
TCGA-G9-6342	FALSE	0.12	-0.36	0.43	-0.44	-0.14	FALSE	-0.19	0.12	0.02	-0.01	0.01
TCGA-M7-A721	FALSE	0.12	-0.16	0.49	-0.06	-0.15	FALSE	0.39	0.12	-0.02	0.01	0.03
TCGA-G9-6336	FALSE	0.11	-0.57	0.62	0.02	0.1	FALSE	0.27	0.11	0	0.05	-0.03
TCGA-ZG-A9LY	FALSE	0.1	-0.54	0.27	-0.25	0.28	FALSE	0.31	0.1	-0.05	0.09	-0.02
TCGA-KK-A6E7	FALSE	0.1	-0.8	0.83	-0.31	-0.95	FALSE	-0.34	0.1	0.07	-0.1	0.03
TCGA-EJ-A7NK	FALSE	0.1	-0.44	0.48	-0.23	-0.32	FALSE	0.97	0.1	-0.01	0.05	-0.02
TCGA-V1-A9O5	FALSE	0.1	1.66	0.12	0.2	0.62	TRUE	0.1	-0.46	0.03	-0.06	-0.04
TCGA-M7-A71Z	FALSE	0.09	-1.29	0.87	-0.11	-0.66	FALSE	-0.37	0.09	0.09	-0.17	0.09
TCGA-HC-7821	FALSE	0.09	1.22	0.15	0.95	-0.26	FALSE	0.44	0.09	-0.06	0.11	-0.04
TCGA-KK-A7AP	FALSE	0.08	-0.34	0.71	-0.15	-0.45	FALSE	-0.6	0.08	0.07	-0.12	0.05
TCGA-EJ-A6RC	FALSE	0.07	-0.03	0.39	0.02	0.23	FALSE	1.67	0.07	-0.06	0.1	-0.02
TCGA-KK-A8IA	FALSE	0.07	0.66	0.56	-0.29	-0.84	FALSE	-1.14	0.07	0.08	-0.1	0.02
TCGA-EJ-7330	FALSE	0.06	-0.42	0.27	0.17	-0.14	FALSE	0.45	0.06	-0.04	0.1	-0.04
TCGA-KC-A7F6	FALSE	0.06	-0.73	0.41	-0.24	0.24	TRUE	0.06	0.4	0.1	-0.06	0.02
TCGA-EJ-A46F	FALSE	0.06	-0.63	0.85	-0.11	0.54	FALSE	-0.57	0.06	0.09	-0.19	0.09
TCGA-G9-6371	FALSE	0.06	0.21	0.71	-0.07	0.08	FALSE	-0.79	0.06	0.05	-0.08	0.03
TCGA-EJ-5527	FALSE	0.05	0.37	0.28	0.09	0.21	FALSE	-0.55	0.05	-0.08	0.11	-0.02
TCGA-YL-A8SQ	FALSE	0.03	-0.06	0.31	0.56	-0.38	FALSE	0.16	0.03	0.01	-0.05	0.04
TCGA-EJ-7331	FALSE	0.02	0.29	0.52	0.28	-0.45	FALSE	-0.15	0.02	-0.09	0.15	-0.04
TCGA-YL-A9WI	FALSE	0.02	0.2	0.68	-0.1	-0.72	FALSE	-0.65	0.02	0.07	-0.1	0.01
TCGA-EJ-7782	FALSE	0.02	0.17	0.39	-0.23	-1.15	FALSE	0.1	0.02	0	0	0
TCGA-EJ-7786	FALSE	0.01	-0.4	0.67	0	-0.48	FALSE	-0.32	0.01	-0.05	0.05	0.01

Patient	Responde	TIDE	IFNG	MSI Score	CD274	CD8	CTL.flag	Dysfunction	Exclusion	MDSC	CAF	TAM M2
TCGA-EJ-7312	FALSE	0.01	-0.72	0.81	-0.23	-0.03	FALSE	-0.06	0.01	0.02	-0.08	0.06
TCGA-V1-A9OL	FALSE	0.01	0.38	0.39	0.12	0.14	FALSE	-0.1	0.01	0.04	-0.05	0.01
TCGA-FC-A66V	FALSE	0.01	-0.28	0.88	-0.23	0.4	FALSE	1.03	0.01	-0.01	-0.02	0.02
TCGA-G9-6339	FALSE	0.01	0.72	0.76	-0.08	0.67	TRUE	0.01	-0.36	0.04	-0.08	-0.02
TCGA-EJ-7314	FALSE	0.01	-0.32	0.58	-0.08	0.08	FALSE	-0.46	0.01	0.08	-0.06	-0.02
TCGA-EJ-A46H	TRUE	0	-1.01	0.15	0	0.56	FALSE	1.51	0	-0.07	0.13	-0.05
TCGA-EJ-5532	TRUE	0	-0.15	0.61	0.12	-0.88	FALSE	-0.83	0	-0.02	0.02	0.01
TCGA-J4-8200	TRUE	-0.01	-0.2	0.73	-0.05	-0.76	FALSE	-0.65	-0.01	-0.06	0.09	-0.02
TCGA-HC-7740	TRUE	-0.01	-0.16	0.37	0.07	0.39	FALSE	1.47	-0.01	-0.06	0.14	-0.07
TCGA-KK-A6E4	TRUE	-0.02	0.56	0.87	0.07	0.08	FALSE	-0.16	-0.02	0.07	-0.13	0.05
TCGA-EJ-5515	TRUE	-0.02	0.33	0.71	0.01	0.23	FALSE	-0.88	-0.02	-0.06	0.07	-0.01
TCGA-VP-A87H	TRUE	-0.02	-0.27	0.58	-0.07	-0.44	FALSE	0.08	-0.02	0.08	-0.07	-0.01
TCGA-HC-7213	TRUE	-0.02	-0.03	0.26	-0.37	-0.26	FALSE	0.12	-0.02	0.04	0	-0.03
TCGA-HC-7749	TRUE	-0.03	0.34	0.45	0.08	-0.14	FALSE	-0.53	-0.03	-0.03	0.02	0
TCGA-VN-A88R	TRUE	-0.03	-0.21	0.73	0.43	-0.92	FALSE	-0.06	-0.03	0.02	-0.08	0.05
TCGA-V1-A8WS	TRUE	-0.04	-0.29	0.47	-0.23	-0.36	FALSE	-1.07	-0.04	0.06	-0.14	0.06
TCGA-V1-A8WV	TRUE	-0.04	0.49	0.06	-0.27	0.6	FALSE	0.34	-0.04	0.04	-0.06	0
TCGA-YL-A8HO	TRUE	-0.04	0.22	0.32	0.17	0.3	FALSE	1.39	-0.04	-0.09	0.08	0
TCGA-G9-7521	TRUE	-0.04	2.53	0.28	0.59	1.48	TRUE	-0.04	-0.58	-0.06	0.06	-0.08
TCGA-VN-A88P	TRUE	-0.04	0.23	0.58	0.14	-0.01	FALSE	-0.09	-0.04	-0.03	0.03	0
TCGA-V1-A9OQ	TRUE	-0.05	-0.33	0.81	-0.1	0.51	TRUE	-0.05	-0.12	-0.01	-0.07	0.05
TCGA-HC-7744	TRUE	-0.06	-0.87	0.66	-0.43	-0.76	FALSE	-1.37	-0.06	0.08	-0.12	0.03
TCGA-ZG-A8QX	TRUE	-0.06	-0.45	0.83	-0.14	-0.22	FALSE	0.03	-0.06	-0.01	0.01	-0.01
TCGA-EJ-A46G	TRUE	-0.06	0.62	0.22	0.28	-0.36	FALSE	0.36	-0.06	0.02	-0.01	-0.03
TCGA-EJ-A8FP	TRUE	-0.07	-0.33	0.37	0.06	0.21	FALSE	0.94	-0.07	-0.07	0.09	-0.03
TCGA-EJ-5497	TRUE	-0.07	-0.3	0.46	-0.01	0.24	FALSE	-0.74	-0.07	-0.06	0.05	0.01
TCGA-G9-6338	TRUE	-0.07	-0.57	0.61	0.12	-0.32	FALSE	0.65	-0.07	-0.02	0.04	-0.02
TCGA-FC-A6HD	TRUE	-0.07	-0.9	0.98	0.2	0.09	FALSE	-0.5	-0.07	0.1	-0.22	0.09
TCGA-M7-A720	TRUE	-0.07	-0.79	0.57	-0.06	-0.08	FALSE	0.74	-0.07	-0.04	0.03	0
TCGA-CH-5766	TRUE	-0.08	0.69	0.45	-0.1	0.32	TRUE	-0.08	-1.4	-0.06	-0.09	-0.06
TCGA-CH-5740	TRUE	-0.08	0.65	0.74	-0.05	0.45	FALSE	-0.86	-0.08	0.06	-0.1	0.03
TCGA-ZG-A9N3	TRUE	-0.09	0.77	0.16	-0.07	0.32	TRUE	-0.09	-0.18	0.11	-0.13	-0.03
TCGA-KK-A8IF	TRUE	-0.09	-0.47	0.62	0.07	-0.94	FALSE	-0.85	-0.09	0.05	-0.12	0.05
TCGA-VP-A87D	TRUE	-0.09	-0.55	0.49	-0.32	-0.38	FALSE	-0.71	-0.09	0.03	-0.1	0.04
TCGA-G9-6343	TRUE	-0.1	-0.74	0.8	0.13	-0.07	FALSE	0.71	-0.1	-0.01	-0.04	0.03
TCGA-KC-A7F5	TRUE	-0.1	-0.27	0.87	-0.05	-0.86	FALSE	-0.66	-0.1	0.08	-0.16	0.05
TCGA-G9-6369	TRUE	-0.1	-0.68	0.84	0.66	-0.45	FALSE	-0.2	-0.1	0.03	-0.08	0.02
TCGA-EJ-5503	TRUE	-0.1	-0.52	0.42	-0.11	0.3	FALSE	1.26	-0.1	-0.06	0.09	-0.04
TCGA-EJ-5526	TRUE	-0.11	0.1	0.31	0.35	-0.12	FALSE	-0.77	-0.11	-0.06	0.1	-0.04
TCGA-KK-A8I9	TRUE	-0.11	-0.12	0.24	0.88	0.27	FALSE	1.02	-0.11	0.02	0.06	-0.1
TCGA-V1-A8WW	TRUE	-0.11	0.61	0.34	-0.19	-0.68	FALSE	0.48	-0.11	0.05	-0.01	-0.06
TCGA-EJ-5521	TRUE	-0.12	0	0.57	-0.14	0.2	FALSE	0.25	-0.12	-0.02	0	0.01
TCGA-HC-A8CY	TRUE	-0.12	1.42	0.63	0.32	0.58	TRUE	-0.12	-1.55	-0.03	-0.16	-0.04
TCGA-HC-8260	TRUE	-0.12	0.47	0.51	-0.03	-0.17	FALSE	0.06	-0.12	-0.03	0	0.01
TCGA-HC-A6AL	TRUE	-0.12	0.31	0.82	-0.18	0.01	FALSE	0.41	-0.12	0.03	-0.09	0.04
TCGA-V1-A8WN	TRUE	-0.13	0.51	0.48	0.17	0.23	TRUE	-0.13	0.04	-0.02	0.07	-0.04
TCGA-QU-A6IN	TRUE	-0.14	-1.36	0.86	0.21	-0.01	FALSE	0.24	-0.14	0.07	-0.16	0.06
TCGA-KC-A7FA	TRUE	-0.14	0.45	0.45	-0.21	0	FALSE	-0.02	-0.14	0.01	-0.03	-0.01
TCGA-M7-A722	TRUE	-0.14	-0.76	0.95	0.54	-0.47	FALSE	-0.08	-0.14	0.05	-0.17	0.08
TCGA-EJ-7325	TRUE	-0.15	-0.96	0.87	0.07	-0.2	FALSE	0.68	-0.15	0.03	-0.11	0.05
TCGA-EJ-A6RA	TRUE	-0.16	-0.1	0.72	-0.07	0.46	FALSE	0.71	-0.16	0.01	-0.08	0.04
TCGA-2A-A8VL	TRUE	-0.16	-0.41	0.74	0	-0.15	FALSE	-0.19	-0.16	-0.01	-0.03	0
TCGA-HC-A6HY	TRUE	-0.17	-0.24	0.76	-0.35	0.39	TRUE	-0.17	-0.05	0.08	-0.16	0.06
TCGA-CH-5788	TRUE	-0.17	-0.2	0.67	0.11	-1.07	FALSE	-1.03	-0.17	0.06	-0.12	0.03
TCGA-EJ-5509	TRUE	-0.17	0.29	0.38	0.31	0.01	FALSE	1.2	-0.17	-0.07	0.06	-0.01
TCGA-YL-A8SH	TRUE	-0.17	-0.51	0.51	-0.31	-0.24	FALSE	0.37	-0.17	0.01	-0.05	0.01
TCGA-YJ-A8SW	TRUE	-0.17	-0.54	0.74	-0.04	-0.74	FALSE	-0.65	-0.17	0.04	-0.08	0.01
TCGA-KK-A7AY	TRUE	-0.18	0.27	0.86	-0.15	0.14	TRUE	-0.18	0.38	0.07	-0.07	0.04
TCGA-CH-5739	TRUE	-0.18	0.95	0.42	-0.12	-0.05	FALSE	-0.44	-0.18	-0.05	0.09	-0.05
TCGA-XA-A8JR	TRUE	-0.18	0.46	0.75	0.24	-0.45	FALSE	0.25	-0.18	-0.02	0.07	-0.08
TCGA-EJ-5517	TRUE	-0.19	0.27	0.62	0.13	0.29	FALSE	-0.75	-0.19	-0.08	0.06	-0.01
TCGA-EJ-7783	TRUE	-0.19	-0.55	0.23	-0.05	0.16	FALSE	0.17	-0.19	-0.02	0.02	-0.02
TCGA-HC-7231	TRUE	-0.19	1.35	0.43	0.02	0.53	TRUE	-0.19	-1.2	-0.06	-0.1	-0.01
TCGA-EJ-5510	TRUE	-0.2	0.66	0.43	0.01	0.56	FALSE	0.57	-0.2	-0.03	0.05	-0.04
TCGA-HC-7212	TRUE	-0.2	0.28	0.27	-0.49	-0.67	FALSE	-0.87	-0.2	0	-0.04	0.02
TCGA-EJ-A46E	TRUE	-0.21	-0.81	0.47	0.26	0.36	FALSE	0.93	-0.21	-0.02	0.06	-0.07
TCGA-XK-AAK1	TRUE	-0.21	-0.02	0.92	0.67	0.22	TRUE	-0.21	-0.84	0.01	-0.19	0.04
TCGA-CH-5746	TRUE	-0.22	-0.78	0.58	-0.26	-0.38	FALSE	-1.09	-0.22	0.02	-0.08	0.02
TCGA-EJ-7789	TRUE	-0.22	-0.59	0.61	-0.19	-1	FALSE	-1.06	-0.22	0.04	-0.15	0.07
TCGA-KK-A59Y	TRUE	-0.23	-0.33	0.54	-0.48	-0.1	FALSE	-0.41	-0.23	0.05	-0.12	0.03

Patient	Responde	TIDE	IFNG	MSI Score	CD274	CD8	CTL.flag	Dysfunction	Exclusion	MDSC	CAF	TAM M2
TCGA-EJ-5494	TRUE	-0.24	0.69	0.41	0.52	0.81	FALSE	0.42	-0.24	-0.02	0	-0.01
TCGA-CH-5762	TRUE	-0.24	0.49	0.33	0.14	0.41	TRUE	-0.24	-0.4	-0.04	0.05	-0.07
TCGA-EJ-A65E	TRUE	-0.24	-0.13	0.79	-0.33	-0.44	FALSE	-0.91	-0.24	0.04	-0.1	0.01
TCGA-CH-5791	TRUE	-0.24	0.2	0.45	-0.14	-0.86	FALSE	-0.64	-0.24	-0.01	-0.02	0
TCGA-VP-A878	TRUE	-0.24	-0.47	0.29	0.17	0.06	FALSE	1.07	-0.24	-0.04	0.04	-0.03
TCGA-EJ-7785	TRUE	-0.25	-0.07	0.21	-0.04	0.3	FALSE	0.47	-0.25	-0.09	0.11	-0.04
TCGA-J4-A83I	TRUE	-0.25	1.22	0.3	0.14	0.04	FALSE	-0.06	-0.25	-0.02	0.02	-0.04
TCGA-CH-5772	TRUE	-0.25	0.92	0.72	0.37	-0.19	FALSE	-1.3	-0.25	0.01	-0.04	-0.01
TCGA-VP-A876	TRUE	-0.25	0.21	0.59	-0.46	0.16	FALSE	-1.28	-0.25	0.09	-0.18	0.05
TCGA-J4-AATV	TRUE	-0.26	-0.72	0.41	-0.05	-0.45	FALSE	1.4	-0.26	-0.05	0.11	-0.09
TCGA-G9-6384	TRUE	-0.26	1.1	0.33	0.14	0.4	FALSE	0.15	-0.26	-0.04	0.07	-0.06
TCGA-KK-A8IB	TRUE	-0.26	-0.2	0.57	0.31	0.54	FALSE	0.81	-0.26	0.02	-0.02	-0.04
TCGA-CH-5794	TRUE	-0.26	-0.29	0.48	-0.31	0.33	FALSE	-0.16	-0.26	0.04	-0.07	-0.01
TCGA-EJ-A65M	TRUE	-0.27	0.15	0.81	-0.08	0.47	TRUE	-0.27	0	0.02	-0.09	0.05
TCGA-KK-A8I6	TRUE	-0.27	-0.01	0.47	-0.18	-0.27	FALSE	-0.53	-0.27	0.04	-0.13	0.04
TCGA-G9-6332	TRUE	-0.29	-0.27	0.78	-0.29	0.57	FALSE	-1.06	-0.29	0.01	-0.15	0.08
TCGA-KK-A6E2	TRUE	-0.29	0.21	0.65	-0.31	-0.2	FALSE	-0.93	-0.29	0.02	-0.13	0.06
TCGA-EJ-5514	TRUE	-0.3	0.61	0.3	0.17	-0.62	FALSE	-0.73	-0.3	-0.04	0.03	-0.02
TCGA-EJ-7125	TRUE	-0.31	0.47	0.7	0.08	-0.15	FALSE	-0.15	-0.31	-0.04	0.02	-0.02
TCGA-CH-5765	TRUE	-0.31	-0.8	0.55	-0.27	-0.88	FALSE	-1.55	-0.31	0.02	-0.08	0.02
TCGA-VN-A88M	TRUE	-0.31	-0.68	0.71	-0.09	0.1	FALSE	-0.32	-0.31	0.06	-0.17	0.05
TCGA-EJ-5519	TRUE	-0.32	-0.42	0.35	-0.3	-0.67	FALSE	-0.13	-0.32	-0.01	-0.02	0
TCGA-HC-8259	TRUE	-0.33	-0.09	0.69	-0.39	-0.14	FALSE	-0.98	-0.33	0.03	-0.14	0.05
TCGA-YL-A8HL	TRUE	-0.34	-0.25	0.4	-0.54	-0.29	FALSE	-0.78	-0.34	0.01	-0.09	0.02
TCGA-HC-7748	TRUE	-0.35	1.17	0.3	0.02	0.2	FALSE	0.32	-0.35	-0.1	0.13	-0.07
TCGA-G9-6333	TRUE	-0.35	1.18	0.64	0.42	-0.02	FALSE	0.68	-0.35	-0.04	0.07	-0.09
TCGA-EJ-A65F	TRUE	-0.35	0.01	0.48	0.12	0.6	TRUE	-0.35	0.06	0	0	0.01
TCGA-HC-8257	TRUE	-0.36	0.3	0.52	0.28	0.67	TRUE	-0.36	-0.05	0.03	-0.09	0.04
TCGA-CH-5741	TRUE	-0.36	-0.51	0.78	-0.25	0.61	FALSE	-1.01	-0.36	0.01	-0.12	0.04
TCGA-HC-8262	TRUE	-0.37	0.18	0.55	-0.18	0.55	FALSE	-0.42	-0.37	0.04	-0.14	0.03
TCGA-TP-A8TV	TRUE	-0.37	-0.83	0.78	-0.02	-0.67	FALSE	-0.57	-0.37	0.05	-0.17	0.05
TCGA-EJ-A65G	TRUE	-0.39	-0.69	0.92	-0.08	-0.64	FALSE	-0.98	-0.39	-0.02	-0.1	0.05
TCGA-MG-AAMC	TRUE	-0.39	0.18	0.8	-0.09	0.06	FALSE	-0.8	-0.39	0.05	-0.15	0.03
TCGA-G9-A9S0	TRUE	-0.39	0.63	0.82	-0.29	0.52	TRUE	-0.39	-0.74	0.03	-0.15	0
TCGA-XJ-A9DX	TRUE	-0.4	-0.57	0.69	0.6	-0.6	FALSE	-0.01	-0.4	0.01	-0.11	0.03
TCGA-VP-A875	TRUE	-0.4	-0.74	0.55	-0.31	-1.14	FALSE	-1.06	-0.4	0.05	-0.18	0.05
TCGA-YL-A8HM	TRUE	-0.4	1.85	0.76	0.34	1.53	TRUE	-0.4	-1	0.01	-0.19	0.01
TCGA-VP-A87J	TRUE	-0.41	-0.07	0.61	-0.43	-0.74	FALSE	-0.66	-0.41	-0.01	-0.01	-0.04
TCGA-KK-A6E8	TRUE	-0.42	-0.89	0.92	0.04	-0.28	FALSE	-1.04	-0.42	0.02	-0.16	0.07
TCGA-QU-A6IL	TRUE	-0.42	-0.8	0.89	0.15	0.14	FALSE	0.57	-0.42	0.04	-0.13	0.02
TCGA-2A-A8VO	TRUE	-0.44	-0.12	0.71	-0.01	-0.27	FALSE	-0.02	-0.44	-0.02	-0.02	-0.03
TCGA-HC-8265	TRUE	-0.44	0.47	0.65	0.51	-0.01	FALSE	0.47	-0.44	0.01	-0.05	-0.03
TCGA-G9-6362	TRUE	-0.46	0.74	0.42	0.67	0.26	FALSE	-0.56	-0.46	0	-0.08	0.01
TCGA-XJ-A83G	TRUE	-0.47	-0.26	0.81	-0.08	-0.14	FALSE	-0.19	-0.47	0.03	-0.16	0.05
TCGA-KK-A6E5	TRUE	-0.47	0.38	0.67	0.14	-0.22	FALSE	0.31	-0.47	-0.04	-0.08	0.04
TCGA-HC-7230	TRUE	-0.48	-0.12	0.57	-0.21	-0.72	FALSE	-1.27	-0.48	0.05	-0.14	0.02
TCGA-EJ-5496	TRUE	-0.48	-0.1	0.7	-0.16	0.22	FALSE	-1.41	-0.48	0	-0.11	0.03
TCGA-KK-A8IJ	TRUE	-0.48	-0.83	0.7	-0.07	-0.74	FALSE	-0.39	-0.48	-0.03	-0.01	-0.02
TCGA-VP-A87B	TRUE	-0.48	-0.68	0.46	0.34	-0.57	FALSE	0.56	-0.48	0	-0.12	0.04
TCGA-ZG-A9LZ	TRUE	-0.49	1.79	0.6	0.14	0.1	TRUE	-0.49	-0.7	0.04	-0.08	-0.07
TCGA-EJ-7794	TRUE	-0.49	-0.32	0.38	-0.06	-0.31	FALSE	0.47	-0.49	-0.1	0.09	-0.05
TCGA-YL-A8SR	TRUE	-0.5	-0.44	0.36	0.27	-0.47	FALSE	-0.48	-0.5	-0.03	-0.07	0.02
TCGA-KK-A8IK	TRUE	-0.5	-1.42	0.5	-0.12	-1	FALSE	-1.03	-0.5	0.06	-0.23	0.08
TCGA-CH-5750	TRUE	-0.5	1.04	0.52	0.2	1.04	TRUE	-0.5	-1.46	0	-0.16	-0.06
TCGA-FC-A5OB	TRUE	-0.51	-0.86	0.96	-0.29	-1.1	FALSE	-1.85	-0.51	0.01	-0.16	0.06
TCGA-EJ-A7NF	TRUE	-0.51	0.15	0.8	-0.17	-0.05	FALSE	-0.69	-0.51	0.05	-0.22	0.08
TCGA-EJ-5508	TRUE	-0.51	0.41	0.31	0.07	0.53	TRUE	-0.51	-0.98	-0.09	-0.02	-0.03
TCGA-HC-7736	TRUE	-0.52	0.01	0.4	0.03	-0.25	FALSE	0.27	-0.52	-0.05	0.03	-0.05
TCGA-EJ-A8FN	TRUE	-0.52	0.15	0.76	-0.05	0.07	FALSE	0.4	-0.52	-0.08	0	0
TCGA-2A-AAYF	TRUE	-0.53	-0.39	0.87	0.01	-0.46	FALSE	-0.79	-0.53	-0.02	-0.09	0.03
TCGA-ZG-A9ND	TRUE	-0.53	-0.18	0.74	0.24	0.14	FALSE	-0.41	-0.53	0.05	-0.14	0
TCGA-J4-A83N	TRUE	-0.53	-0.06	0.73	-0.37	-0.31	FALSE	-0.6	-0.53	0.01	-0.12	0.02
TCGA-HC-7209	TRUE	-0.53	0.09	0.57	-0.22	-0.02	FALSE	-0.18	-0.53	-0.01	0	-0.06
TCGA-EJ-5505	TRUE	-0.54	-0.49	0.76	0.21	-0.04	FALSE	-1.17	-0.54	-0.03	-0.1	0.05
TCGA-VP-A87K	TRUE	-0.55	0.34	0.36	-0.21	0.62	FALSE	-0.51	-0.55	0.05	-0.2	0.05
TCGA-HC-7738	TRUE	-0.56	-0.11	0.69	-0.06	-0.42	FALSE	-0.89	-0.56	0	-0.12	0.03
TCGA-EJ-7321	TRUE	-0.56	1.29	0.67	0.12	0.47	TRUE	-0.56	-1.17	-0.02	-0.15	-0.01
TCGA-G9-6366	TRUE	-0.57	0.73	0.76	-0.16	0.19	TRUE	-0.57	-0.55	-0.02	-0.08	0.01
TCGA-KK-A6E1	TRUE	-0.57	0.42	0.43	-0.2	-0.78	FALSE	-0.7	-0.57	0.07	-0.13	-0.04
TCGA-EJ-5506	TRUE	-0.57	0.69	0.56	-0.05	-0.14	FALSE	-0.45	-0.57	-0.04	-0.01	-0.03

Patient	Responde	TIDE	IFNG	MSI Score	CD274	CD8	CTL.flag	Dysfunction	Exclusion	MDSC	CAF	TAM M2
TCGA-CH-5767	TRUE	-0.57	-0.7	0.87	0.21	-0.73	FALSE	-0.89	-0.57	-0.03	-0.04	-0.02
TCGA-EJ-7784	TRUE	-0.58	0.45	0.52	-0.24	-0.32	FALSE	-0.91	-0.58	0.02	-0.13	0.02
TCGA-VN-A88Q	TRUE	-0.59	-0.49	0.38	-0.31	-0.41	FALSE	-0.48	-0.59	0.02	-0.1	-0.01
TCGA-M7-A724	TRUE	-0.6	-1.37	0.96	-0.06	-0.99	FALSE	-0.72	-0.6	0.09	-0.26	0.06
TCGA-V1-A8WL	TRUE	-0.6	0.7	0.49	0.18	0.43	FALSE	0.67	-0.6	-0.06	0.03	-0.06
TCGA-EJ-7797	TRUE	-0.6	0.49	0.34	-0.03	0.08	FALSE	-0.22	-0.6	-0.01	-0.05	-0.03
TCGA-G9-6499	TRUE	-0.6	-0.43	0.78	0.46	0.32	FALSE	-0.45	-0.6	0	-0.07	-0.03
TCGA-M7-A725	TRUE	-0.61	0.78	0.45	-0.04	-0.72	FALSE	-0.88	-0.61	0.06	-0.1	-0.05
TCGA-G9-A9S7	TRUE	-0.62	0.06	0.68	-0.27	0.27	TRUE	-0.62	-0.47	0.06	-0.19	0.04
TCGA-CH-5768	TRUE	-0.63	-0.11	0.63	-0.04	-0.09	FALSE	-1.46	-0.63	0.02	-0.13	0.02
TCGA-EJ-A65J	TRUE	-0.63	0.21	0.6	-0.04	-0.84	FALSE	-0.65	-0.63	-0.06	-0.04	0
TCGA-KC-A7FD	TRUE	-0.64	-0.39	0.63	0.18	-0.42	FALSE	-0.97	-0.64	0.05	-0.14	-0.02
TCGA-VN-A88N	TRUE	-0.66	0.14	0.78	-0.32	-0.07	FALSE	-0.69	-0.66	0.02	-0.15	0.03
TCGA-EJ-5516	TRUE	-0.67	1.59	0.41	0.4	0.14	TRUE	-0.67	-0.3	-0.07	0.1	-0.06
TCGA-EJ-7123	TRUE	-0.68	-0.6	0.6	0	-0.52	FALSE	-0.48	-0.68	-0.02	-0.1	0.02
TCGA-XK-AAJP	TRUE	-0.68	0.39	0.67	0.02	-0.67	FALSE	-0.3	-0.68	0	-0.13	0.02
TCGA-J4-AAU2	TRUE	-0.68	-0.18	0.78	0.04	0.05	FALSE	0.12	-0.68	-0.02	-0.09	0.01
TCGA-G9-6354	TRUE	-0.68	0.45	0.8	-0.1	0.65	FALSE	-0.18	-0.68	0	-0.14	0.02
TCGA-YL-A8S9	TRUE	-0.68	-1.07	0.87	-0.09	-0.99	FALSE	-1.51	-0.68	0.04	-0.22	0.07
TCGA-EJ-5499	TRUE	-0.69	0.99	0.54	0.17	0.35	TRUE	-0.69	0.28	0.07	-0.04	0.01
TCGA-J4-AATZ	TRUE	-0.69	0.29	0.58	1.25	-0.39	FALSE	-0.62	-0.69	0.01	-0.1	-0.02
TCGA-CH-5764	TRUE	-0.69	0.74	0.49	0.13	0.8	TRUE	-0.69	-0.65	-0.02	-0.03	-0.04
TCGA-EJ-7218	TRUE	-0.71	0.3	0.74	0.12	-0.89	FALSE	0.17	-0.71	-0.02	-0.12	0.03
TCGA-EJ-8468	TRUE	-0.71	0.15	0.42	0.12	0.07	FALSE	0.16	-0.71	-0.13	0.08	-0.05
TCGA-EJ-7781	TRUE	-0.72	0.01	0.37	-0.04	0.01	FALSE	-0.04	-0.72	-0.08	0.01	-0.03
TCGA-KK-A8IH	TRUE	-0.72	0.71	0.3	-0.28	-0.63	FALSE	0.08	-0.72	0	-0.08	-0.03
TCGA-KK-A6E0	TRUE	-0.73	0.11	0.74	0.04	-0.62	FALSE	-0.87	-0.73	0.04	-0.19	0.03
TCGA-EJ-5502	TRUE	-0.73	0.15	0.28	-0.07	-0.18	FALSE	0.92	-0.73	-0.09	0.08	-0.09
TCGA-HC-7819	TRUE	-0.74	-0.01	0.67	-0.22	-0.69	FALSE	-1.41	-0.74	0.02	-0.15	0.02
TCGA-XJ-A83H	TRUE	-0.75	0.04	0.71	-0.01	0.12	FALSE	0.11	-0.75	0.02	-0.06	-0.07
TCGA-HC-7077	TRUE	-0.75	0.14	0.79	-0.24	-0.24	FALSE	-0.95	-0.75	0.04	-0.17	0.01
TCGA-YL-A9WJ	TRUE	-0.75	0	0.51	-0.05	-0.27	FALSE	0.89	-0.75	-0.08	0.04	-0.06
TCGA-XK-AAIW	TRUE	-0.76	1.23	0.9	0.07	2.17	TRUE	-0.76	-0.7	0.1	-0.24	0.03
TCGA-J9-A52D	TRUE	-0.76	-0.85	0.94	0.2	-0.91	FALSE	-1.35	-0.76	0.04	-0.21	0.05
TCGA-J4-A67T	TRUE	-0.76	0.35	0.5	0.62	0.27	FALSE	0.3	-0.76	-0.03	0.04	-0.11
TCGA-EJ-5522	TRUE	-0.77	1.08	0.41	0.23	0.3	TRUE	-0.77	-0.38	-0.05	0.01	-0.01
TCGA-KK-A7AU	TRUE	-0.79	0.17	0.67	-0.07	-0.13	FALSE	-0.7	-0.79	0.05	-0.22	0.03
TCGA-CH-5792	TRUE	-0.8	0.84	0.19	0.28	0.54	FALSE	1.81	-0.8	-0.12	0.12	-0.1
TCGA-KK-A8II	TRUE	-0.81	-0.01	0.65	-0.25	-0.27	FALSE	-1.12	-0.81	0.05	-0.17	-0.01
TCGA-KK-A8ID	TRUE	-0.82	1.57	0.79	0.22	-0.31	FALSE	-0.85	-0.82	0.03	-0.17	0.02
TCGA-HC-8258	TRUE	-0.82	0.18	0.66	0.19	-0.03	FALSE	0.87	-0.82	-0.04	-0.05	-0.04
TCGA-EJ-5507	TRUE	-0.83	1.96	0.35	0.53	0.19	TRUE	-0.83	-0.49	-0.01	-0.04	-0.02
TCGA-J4-A83M	TRUE	-0.85	0.42	0.58	-0.13	0.43	FALSE	0.69	-0.85	-0.04	-0.05	-0.04
TCGA-J9-A8CN	TRUE	-0.86	-0.91	0.86	0.29	-0.27	FALSE	-0.29	-0.86	-0.01	-0.1	-0.01
TCGA-HC-8216	TRUE	-0.88	1.34	0.65	0.55	0.38	TRUE	-0.88	-0.73	-0.01	-0.07	-0.03
TCGA-HC-7820	TRUE	-0.88	0.58	0.62	-0.02	0.33	FALSE	-0.37	-0.88	-0.03	-0.12	0.01
TCGA-CH-5738	TRUE	-0.89	-0.06	0.37	0	0.12	FALSE	0.45	-0.89	-0.13	0.11	-0.1
TCGA-2A-A8VT	TRUE	-0.89	0.62	0.26	-0.02	0.38	FALSE	-0.34	-0.89	0	-0.12	-0.03
TCGA-Y6-A9XI	TRUE	-0.9	-0.68	0.59	-0.31	-0.62	FALSE	-0.43	-0.9	-0.03	-0.16	0.05
TCGA-HC-8256	TRUE	-0.92	0.42	0.82	-0.14	0.08	FALSE	-1.03	-0.92	0	-0.19	0.04
TCGA-WW-A8ZI	TRUE	-0.93	0.22	0.59	0.31	-0.17	FALSE	-0.46	-0.93	-0.04	-0.12	0.02
TCGA-CH-5748	TRUE	-0.94	0.22	0.74	0.12	-0.24	FALSE	-1.39	-0.94	0	-0.14	0
TCGA-HC-7818	TRUE	-0.94	-0.05	0.42	-0.18	-0.11	FALSE	0.17	-0.94	-0.07	0	-0.06
TCGA-2A-AAYO	TRUE	-0.95	0.93	0.61	0.3	0.59	FALSE	1.01	-0.95	-0.09	-0.02	-0.03
TCGA-KC-A4BN	TRUE	-0.95	-0.03	0.69	0.25	0.1	FALSE	-0.45	-0.95	0.01	-0.09	-0.06
TCGA-HC-A6AO	TRUE	-0.95	0.69	0.89	0.14	-0.17	FALSE	-0.06	-0.95	0.01	-0.17	0
TCGA-HC-8213	TRUE	-0.97	-0.3	0.91	-0.13	-0.49	FALSE	-1.91	-0.97	0	-0.21	0.05
TCGA-CH-5737	TRUE	-0.98	-0.21	0.74	1.32	-0.77	FALSE	-0.23	-0.98	-0.04	-0.11	0
TCGA-CH-5789	TRUE	-1.02	-0.74	0.49	-0.07	-0.14	FALSE	-0.01	-1.02	-0.13	0.06	-0.08
TCGA-KK-A6DY	TRUE	-1.03	0.81	0.35	-0.04	0.26	FALSE	-0.07	-1.03	-0.05	-0.01	-0.1
TCGA-HC-7080	TRUE	-1.03	-0.13	0.65	0.26	-0.93	FALSE	-0.12	-1.03	0.01	-0.2	0.02
TCGA-VN-A943	TRUE	-1.07	-0.85	0.97	1.21	-0.55	FALSE	-1.14	-1.07	0.01	-0.22	0.03
TCGA-XK-AAJA	TRUE	-1.09	-0.46	0.38	0.16	-0.55	FALSE	0.26	-1.09	-0.01	-0.16	0
TCGA-HC-7752	TRUE	-1.09	-1.05	0.5	0.02	-0.12	FALSE	1.39	-1.09	-0.06	-0.14	0.03
TCGA-CH-5790	TRUE	-1.1	-0.34	0.37	-0.19	-0.35	FALSE	-1.01	-1.1	-0.06	-0.09	-0.01
TCGA-EJ-A8FS	TRUE	-1.11	-0.43	0.86	0.24	-0.15	FALSE	-0.58	-1.11	0.01	-0.22	0.03
TCGA-2A-A8W1	TRUE	-1.14	-0.41	0.94	0.09	-0.49	FALSE	-1.49	-1.14	0.02	-0.22	0.02
TCGA-YL-A8SA	TRUE	-1.19	0.66	0.6	-0.13	-0.65	FALSE	-0.43	-1.19	-0.01	-0.14	-0.03
TCGA-J4-A83J	TRUE	-1.19	0.42	0.63	0.37	-0.01	FALSE	0.21	-1.19	-0.02	-0.09	-0.08
TCGA-V1-A9OF	TRUE	-1.2	-0.33	0.73	0.22	-0.11	FALSE	-0.77	-1.2	-0.03	-0.23	0.07

Patient	Responde	TIDE	IFNG	MSI Score	CD274	CD8	CTL.flag	Dysfunction	Exclusion	MDSC	CAF	TAM M2
TCGA-VN-A88O	TRUE	-1.21	0.83	0.81	0.82	0.17	FALSE	0.24	-1.21	-0.02	-0.09	-0.08
TCGA-CH-5744	TRUE	-1.23	0.23	0.45	-0.23	0.18	FALSE	0.63	-1.23	-0.05	-0.08	-0.04
TCGA-HC-7078	TRUE	-1.24	-0.15	0.38	0.12	-0.15	FALSE	0.55	-1.24	-0.06	-0.1	-0.02
TCGA-EJ-A7NN	TRUE	-1.31	0.24	0.33	0.01	-0.23	FALSE	1	-1.31	-0.03	-0.08	-0.08
TCGA-FC-A4JI	TRUE	-1.36	0.98	0.91	-0.09	0.8	TRUE	-1.36	-0.72	0.1	-0.23	0.02
TCGA-HC-7211	TRUE	-1.4	-0.61	0.41	0.98	-0.71	FALSE	-0.23	-1.4	-0.07	-0.03	-0.1
TCGA-HC-7075	TRUE	-1.55	0.6	0.86	0.07	-0.48	FALSE	-0.36	-1.55	-0.06	-0.15	-0.03
TCGA-CH-5753	TRUE	-1.76	1.61	0.39	0.51	-0.45	FALSE	-0.56	-1.76	-0.04	-0.11	-0.12
